# Cytokines and Immune Cell Phenotype in Acute Kidney Injury Associated With Immune Checkpoint Inhibitors

**DOI:** 10.1016/j.ekir.2022.11.020

**Published:** 2022-12-05

**Authors:** Naba Farooqui, Mark Zaidi, Lisa Vaughan, Trevor D. McKee, Eram Ahsan, Kevin D. Pavelko, Jose C. Villasboas, Svetomir Markovic, Timucin Taner, Nelson Leung, Haidong Dong, Mariam P. Alexander, Sandra M. Herrmann

**Affiliations:** 1 Division of Nephrology and Hypertension, Mayo Clinic, Rochester, Minnesota, USA; 2 Department of Medical Biophysics, University of Toronto, Ontario, Canada; 3 Quantitative Health Sciences, Mayo Clinic, Rochester, Minnesota, USA; 4 Deciphex Inc., Chicago, Illinois, USA; 5 Department of Immunology, Mayo Clinic College of Medicine and Science, Rochester, Minnesota, USA; 6 Division of Hematology, Mayo Clinic, Rochester, Minnesota, USA; 7 Department of Oncology, Mayo Clinic, Rochester, Minnesota, USA; 8 Department of Surgery, Mayo Clinic, Rochester, Minnesota, USA; 9 Department of Laboratory Medicine and Pathology, Mayo Clinic, Rochester, Minnesota, USA

**Keywords:** acute interstitial nephritis, acute kidney injury, biomarkers, cytokines, immune cell phenotyping, immune checkpoint inhibitors

## Abstract

**Introduction:**

Immune checkpoint inhibitors (ICIs) induce impressive antitumor responses but may lead to acute kidney injury (AKI) associated with ICI therapy (AKI-ICI). Biomarkers distinguishing AKI-ICI from AKI because of other causes (AKI-other) are currently lacking. Because ICIs block immunoregulatory pathways, we hypothesized that biomarkers related to immune cell dysregulation, including tumor necrosis factor alpha (TNF-α) and other markers of B and T cell activation in the systemic circulation and kidney tissue, may aid with the diagnosis of AKI-ICI.

**Methods:**

This is a prospective study consisting of 24 participants who presented with AKI during ICI therapy, adjudicated to either have AKI-ICI (*n* = 14) or AKI-other (*n* = 10). We compared markers of kidney inflammation and injury (neutrophil gelatinase-associated lipocalin, kidney injury molecule-1) as well as plasma and urine levels of T cell-associated cytokines (TNF-α, interferon-γ, interleukin (IL)-2, IL-4, IL-6, IL-8, IL-9, and IL-10) between groups. We also compared T-cell responses in the systemic circulation and in kidney tissue across groups, using mass cytometry systems.

**Results:**

We observed increase in several specific immune cells, including CD4 memory, T helper cells, and dendritic cells in the kidney tissue, as well as in the urine cytokines IL-2, IL-10, and TNF-α, in patients who developed AKI-ICI compared to patients with AKI-other (*P* < 0.05 for all). The discriminatory ability of TNF-α on AKI cause was strong (area under the curve = 0.814, 95% confidence interval: 0.623–1.00. The CD4+ T cells with memory phenotype formed the dominant subset.

**Conclusion:**

These results suggest that specific T-cell responses and their respective cytokines may be indicative of AKI associated with ICI therapy and may help to differentiate AKI-ICI from AKI-other. Urine TNF-α is a promising biomarker for AKI-ICI, which is most often caused by acute interstitial nephritis (AIN), and TNF-α pathway may serve as a potential target for therapeutic intervention.

## Graphical abstract

**Figure:**
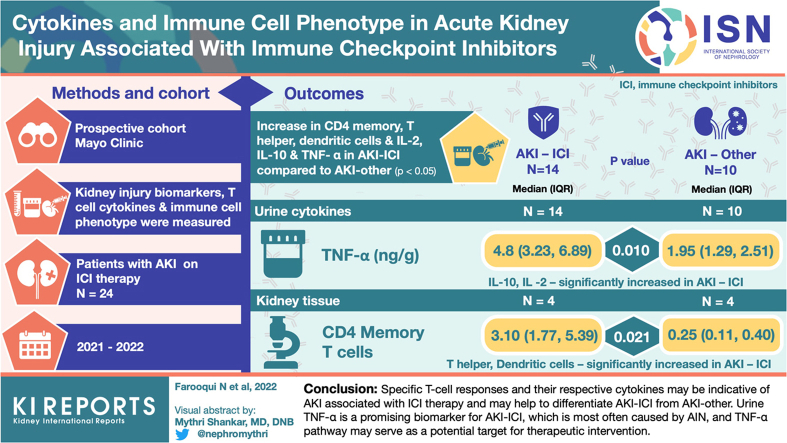

ICIs have improved progression-free and overall survival of many patients with different types of cancer.^1^ With recent studies demonstrating the therapeutic benefit of ICIs either as a single agent or in combination with other ICIs or nephrotoxic cancer agents (e.g., platinum or vascular endothelial growth factor inhibitors), ICI therapies are being used more frequently. However, these therapies are known to induce inflammatory tissue damage, causing immune-related adverse events (irAEs)^2^ which can occur in up to 80% of patients treated with ICI.^3^ Overall, incidence of AKI in patients receiving immunotherapy can reach up to 17%, with 2% to 5% estimated to be directly attributable to immunotherapy.^4^^,^^5^ Programmed cell death protein 1 or programmed cell death ligand 1 signaling pathway, or the cytotoxic T lymphocyte antigen 4 signaling pathway blockade by monoclonal antibodies breaks immune tolerance by unleashing quiescent tissue-specific self-reactive T cells, leading to T-cell dysregulation and development of irAEs. In the kidney, AIN is the most common histopathological finding in patients with AKI-ICI, occurring in 80% to 90% of the cases.^6^, ^7^, ^8^ Patients with AKI-ICI as part of irAEs may present with increased levels of cytokines correlating with T cell activation.

Severe AKI-ICI can be life threatening and may lead to prolonged hospitalization and other morbidities, including chronic kidney disease (CKD).^9^ Although most of the severe irAEs can be reversed with high-dose steroids and/or other immunosuppressive therapies, they often require prolonged courses of immunosuppression, which may lead to complications, thereby increasing adverse events.^10^ Therefore, early recognition of AKI-ICI allows for prompt initiation of immunosuppressive treatments, resulting in reduced toxicity, reduced need for vigorous immunosuppression, as well as preventing the progression of AKI to more severe grades (3−4).^11^ Therefore, the identification of biomarkers associated with AKI-ICI in patients receiving ICI therapies who had AKI events would greatly assist with noninvasive diagnosis and facilitating clinical management, with the potential to improve patient outcomes.

We have previously shown that patients with AIN induced by ICI therapy present with increased blood inflammatory markers such as C-reactive protein and elevated markers of tubular injury such as urine retinol binding protein-to-creatinine ratio.^5^ However, these biomarkers require external validation. Moledina *et al.*^12^ have shown that urine TNF-α and IL-9 improve discrimination over clinician prebiopsy diagnosis of AIN and could be helpful in the setting of ICI-AKI, but these biomarkers have not been validated in the setting of ICI therapy.^12^ Kidney biopsy is currently the gold standard for diagnosing AKI-ICI; however, it is an invasive procedure and may lead to major complications, ranging from 1% to 4% in hospitalized patients.^13^^,^^14^ Thus, identifying AKI-ICI without requiring a biopsy could help inform the clinician about whether further interventions are needed. Furthermore, elucidating pathobiological mechanisms of AKI-ICI during the process of immune cell dysregulation and their association with respective T cell cytokines can offer guidance on specific direct immunosuppressive therapy to reduce kidney injury without jeopardizing the effect of immunotherapy on the cancer being treated.

In this current study, we aimed to investigate blood and urine cytokines and immune cell phenotypes in the peripheral blood and kidney tissue of patients on ICI therapy at the time of AKI to differentiate AKI-ICI from AKI-other.

## Methods

### Study Design and Population

Patients receiving ICI therapy and referred for nephrology consultation with suspicion of AKI-ICI were prospectively enrolled between April 2021 and April 2022. Patients who had blood, urine, and/or kidney biopsy data available at the time of AKI were included in this study. Blood and urine from kidney donors obtained before kidney donation and time zero implantation kidney biopsies were also collected and used as healthy controls. ICIs were defined as the following: cytotoxic T lymphocyte antigen 4 inhibitors (ipilimumab), programmed cell death protein 1 inhibitors (pembrolizumab, nivolumab, and cemiplimab), and programmed cell death ligand 1 antibodies (atezolizumab, avelumab, and durvalumab). Patients who did not provide research authorization were excluded. This study was approved by Mayo Clinic Institutional Review Board.

### Data Collection

Demographic characteristics, kidney function, proteinuria, and medication history at presentation were recorded via manual chart review. Baseline creatinine level was defined as the last stable serum creatinine value before initiating ICI therapy in patients or kidney donation in the control group. AKI events were defined as a ≥1.5-fold increase in serum creatinine level from baseline or an increase of ≥0.3 mg/dl (grade 1 kidney toxicity).^15^ AKI cases directly attributable to other recognizable reasons (e.g., obstruction, sepsis, or systemic hemodynamic changes) or those that did not meet AKI criteria (see Supplementary Table S1) were excluded from analysis. AKI events and their likely causes, including AKI-ICI, were identified either by kidney biopsy-confirmed AIN or by kidney function responsiveness to steroids or progression without steroids, which was determined on the basis of clinical evaluation by the consulting nephrologist at the time of the clinical event. Patients considered to have AKI not related to ICI (AKI-other) were either biopsy-confirmed alternative causes or did not receive steroids and did not progress (Supplementary Table S1). If a determination was unclear, the diagnosis was confirmed by mutual consensus by the authors (SH and NL). Biomarkers were not part of the adjudication process to distinguish AKI-ICI from AKI-other. Measures of kidney function (serum creatinine level and estimated glomerular filtration rate, estimated using the CKD Epidemiology Collaboration equation), as well as the clinical biomarkers C-reactive protein and urine retinol binding protein-to-creatinine ratio were also collected at the time of the AKI event or time of kidney donation if applicable. AKI severity was staged according to the Kidney Disease Improving Global Outcomes Work Group criteria.^16^ Renal recovery was defined as return of kidney function back to baseline or <25% from baseline at 3 months. Blood and urine for plasma and urine cytokines, respectively, as well as peripheral blood monocytes cells (PBMCs) were obtained from all patients if available at the time of AKI and subsequently for a subset of patients that returned for follow-up during taper of corticosteroids. Twelve kidney donors’ urine, plasma and kidney biopsy samples were obtained from another Mayo Clinic Institutional Review Board approved study biorepository.

### Peripheral Blood Mononuclear Cells Isolation

Peripheral blood samples were obtained from patients at the time of AKI. All samples were collected at Mayo Clinic’s central laboratory and PBMCs were processed in the research laboratory. PBMCs were isolated using density-gradient centrifugation (Ficoll-Paque solution) (GE Healthcare Biosciences) and cells were then slowly frozen to −80 °C and subsequently to less than −196 °C in liquid nitrogen for batch analysis.

### PBMC Immune Cell Phenotyping

Among patients with an AKI event, patient derived PBMCs were isolated from whole blood by Ficoll-Hypaque density-gradient centrifugation and cryopreserved in fetal bovine serum (Thermo Fisher cat# 26140079) with 10% dimethyl sulfoxide (Sigma Aldrich cat# D2650). Stored samples were thawed for acquisition in a single batch to minimize variability. Samples were stained according to a previously established protocol developed at the Mayo Clinic Immune Monitoring Core with an antibody panel focusing on cell surface antigens (Supplementary Table S2).^17^ Custom antibodies were generated using the Maxpar Direct X8 antibody labeling kit (Fluidigm, San Francisco).^18^ Data from samples were acquired on the Helios mass cytometry system (Fluidigm, San Francisco). Single-cell data were clustered using the FlowSOM R package^19^ and labeled using the Ek’Balam algorithm.^20^ Cell subset definitions follow Maecker *et al.*,^21^ and Finak *et al.*^22^ Cluster labeling, method implementation, and visualization were done through the Astrolabe Cytometry Platform (Astrolabe Diagnostics, Inc., Fort Lee, NJ).

### Imaging Mass Cytometry Methods

All tissue staining and slide preparation was performed by the Mayo Clinic Pathology Research Core. Please see the imaging mass cytometry methods section in the Supplementary materials including Supplementary Tables S4 and S5 for further details.

### Plasma and Urine Cytokines

Collected blood and urine were transferred to the research laboratory and used to measure cytokines from plasma and urine samples. They were aliquoted and stored at −80 °C and thawed preceding the experiments. Urine and plasma samples were collected at the time of the diagnosis of AKI or within 7 days of the kidney biopsy (when clinically indicated). We used the manufacturer validated MILLIPLEX MAP Human Cytokine (Millipore, Billerica, MA) customized proinflammatory panel to test plasma and urine TNF-α, interferon-gamma, IL-2, IL-4, IL-6, IL-8, IL-9, and IL-10, catalog number # HCYTA-60K. We normalized all the urine cytokines to urine creatinine to account for urine concentration differences using a creatinine parameter assay kit, catalog number #KGE005. The detection ranges of each assay are outlined in Supplementary Table S3.

### Urine Markers of Kidney Injury

Human urine neutrophil gelatinase-associated lipocalin was tested by enzyme-linked immunosorbent assay according to the manufacturer’s protocol (catalog number: KIT 036; BioPorto Diagnostics). Human urine kidney injury molecule-1 was tested according to the manufacturer’s protocol (catalog number: DKM 100; R&D Systems).

### TNF-α Staining of Biopsy Specimens

We performed immunohistochemistry staining for TNF-α to evaluate expression in the kidney tissue to confirm results of the multiplex assay. Briefly, formalin-fixed paraffin-embedded sections were deparaffinized and rehydrated. Antigen retrieval was performed using Dako Target retrieval (Catalog number: S1700) and slides were stained with TNF-α (Abcam, catalog number: ab6771) using Vector Labs Vectastain Elite ABC HRP kit (catalog number: PK6101) according to manufacturer’s instructions. These slide images were captured with ZEISS Axio Observer and edited in ZEN software (ZEISS, Munich Germany). An image-analysis code in MATLAB (MathWorks, Natick, MA) was used to estimate the percentage TNF-α based on the intensity and area of staining (identified by the program from the amount of brown signal in the t image). The staining intensity therefore was semiautomatically quantified in 15 to 20 fields and expressed as a percentage of the slide stained. Results from all fields were then averaged per patient.

### Statistical Methods

Summary statistics were presented as mean (SD) for continuous normally distributed variables, median (interquartile range) for continuous variables with skewed distributions, and as *n* (%) for categorical variables. Comparisons of measures across AKI groups were evaluated using the equal variance *t*-test for normally distributed variables, the Wilcoxon rank sum test for non-normally distributed variables, the χ^2^ test for categorical variables where the expected cell counts were >5, and the Fisher exact test for categorical variables where the expected cell counts were <5. The discrimination between AKI-ICI and AKI on the basis of log-transformed TNF-α levels was evaluated based on area under the curve index estimates derived from a receiver operating characteristic curve fit using logistic regression, with 95% confidence intervals derived using the DeLong method. Correlations were evaluated using Spearman’s rho. Values below the limits of detection were imputed by dividing the minimum detection limit by 2. All analyses were performed using SAS version 9.4 (SAS Institute Inc., Cary, NC) and R version 4.1.2 (R Foundation for Statistical Computing, Vienna, Austria). All *P*-values were 2-tailed and were considered statistically significant at the 0.05 α level.

## Results

### Patient Population and Clinical Characteristics

**Figure 1:**
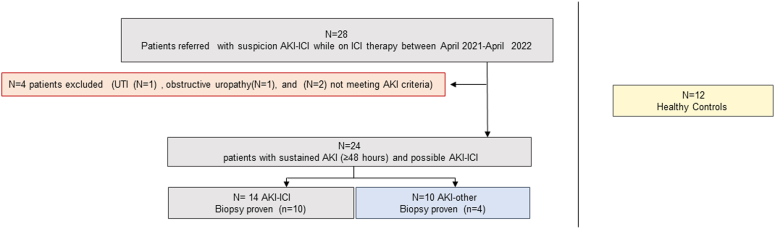
Flowchart of cohort inclusion/exclusion criteria. Flowchart of patients with possible AKI-ICI referred for nephrology consultation and causes of exclusion. All patients with suspicion AKI-ICI were initially enrolled (*N* = 28). After enrollment, 4 patients were subsequently excluded (2 did not meet criteria due nonsustained AKI and 2 were excluded because of subclinical UTI and progressive obstructive uropathy). A total of 24 patients were analyzed. AKI, acute kidney injury; AKI-ICI, AKI associated to immune checkpoint inhibitor; AKI-other, AKI associated to other causes; UTI, urinary tract infection.

During the study period from April 1, 2021 to April 1, 2022, a total of 28 patients were referred to the Nephrology Division at Mayo Clinic Rochester with suspected AKI associated with ICI therapy. All patients referred were enrolled in the study for blood and urine collection. Among these patients, 4 (14%) had AKIs that were directly attributable to non-ICI causes (e.g., obstruction, infection) or did not meet criteria for sustained AKI and were excluded from further analysis. A total of 24 patients remained that had possible ICI-related AKI. Of these, 14 (58%) patients had clinically suspected or biopsy-proven AKI-ICI, whereas the remaining 10 (42%) had other causes for AKI. Twelve healthy kidney donors from another internal Institutional Review Board approved study were also used as controls (see Figure 1).

Baseline characteristics for the *n* = 24 patients with AKI are presented in Table 1 and for all groups including controls in Supplementary Table 11. Age, gender, race, and kidney function were similar among patients across AKI groups. Clinical characteristics, comorbidities, and medications between AKI groups are presented in Table 2. Rates of comorbidities and autoimmune disease were similar across groups, with the most common malignancies being lung cancer, bladder or urothelial cancer, and melanoma. Patients with AKI-ICI had higher rates of tubulo-interstitial nephritis drugs administered at the time of AKI compared to AKI-other patients (8 [57.1%] vs. 1 [10.0%] respectively, *P* = 0.019). There were no significant differences observed in overall irAE rates or type of ICI between the 2 groups. In addition, there were no significant differences observed in administration of other cancer therapeutic agents (e.g., platinum, and vascular endothelial growth factor signaling pathways inhibitor agents) within 1 month before the AKI diagnosis. Renal recovery tended to occur more frequently in the AKI-ICI group compared to the AKI-other group, but results were not statistically significant.

**Table 1:** Demographic characteristics and laboratory measurements, by AKI cause

Variable	AKI-ICI (*N* = 14)	AKI-Other (*N* = 10)	Total (*N* = 24)	*P*-value
Age (yr), mean (SD)	65.4(14.6)	62.4(14.2)	64.1(14.2)	0.63
Gender				0.63
Male	7(50.0%)	6(60.0%)	13(54.2%)	
Female	7(50.0%)	4(40.0%)	11(45.8%)	
White race, n (%)	14(100.0%)	10(100.0%)	24(100.0%)	-
Serum creatinine, median (IQR)	1.9 (1.7,2.4)	1.7 (1.5, 3.5)	1.8 (1.6, 2.6)	0.62
eGFR (CKD-EPI), mean (SD)	29.9(10.8)	34.0(17.9)	31.6(14.0)	0.49

AKI, acute kidney injury; CKD-EPI, chronic kidney disease-epidemiology collaboration equation; eGFR, estimated glomerular filtration rate; ICI, immune checkpoint inhibitors; IQR, interquartile range.

*P*-values are derived from the equal variance *t*-test for normally distributed variables, the Wilcoxon rank sum test for non-normally distributed variables, the χ^2^ test for categorical variables where the expected call counts were ≥5, and the Fisher exact test for categorical variables with an expected cell count of <5.

**Table 2:** Clinical characteristics, by AKI cause

Variable	AKI-ICI (*N* = 14)	AKI-Other (*N* = 10)	Total (*N* = 24)	*P*-value
HTN, n (%)	9 (64.3%)	5 (50.0%)	14 (58.3%)	0.48
DM, n (%)	4 (28.6%)	2 (20.0%)	6 (25.0%)	>0.99
CKD, n (%)	4 (28.6%)	4 (40.0%)	8 (33.3%)	0.67
COPD, n (%)	4 (28.6%)	1 (10.0%)	5 (20.8%)	0.36
History of autoimmune disease, n (%)				
T1 DM	0 (0.0%)	1 (10.0%)	1 (4.2%)	0.42
Hashimoto’s thyroiditis	0 (0.0%)	1 (10.0%)	1 (4.2%)	0.42
IBD	0 (0.0%)	1 (10.0%)	1 (4.2%)	0.42
Malignancy treated with ICI, n (%)				0.71
Missing	2 (14.3%)	1 (10.0%)	3 (12.5%)	
Melanoma	6 (42.9%)	2 (20.0%)	8 (33.3%)	
Lung adenocarcinoma	1 (7.1%)	0 (0.0%)	1 (4.2%)	
Lung squamous cell	2 (14.3%)	3 (30.0%)	5 (20.8%)	
Renal cell	0 (0.0%)	1 (10.0%)	1 (4.2%)	
Bladder/Urothelial	3 (21.4%)	3 (30.0%)	6 (25.0%)	
ICI’s received within 8 wks before first AKI episode, n (%)				
Ipilimumab (CTLA-4)	2 (14.3%)	1 (10.0%)	3 (12.5%)	>0.99
Tremelimumab (CTLA-4)	0 (0.0%)	0 (0.0%)	0 (0.0%)	^-^
Nivolumab (PD-1)	2 (14.3%)	4 (40.0%)	6 (25.0%)	0.19
Pembrolizumab (PD-1)	9 (64.3%)	4 (40.0%)	13 (54.2%)	0.41
Atezolizumab (PD-L1)	2 (14.3%)	1 (10.0%)	3 (12.5%)	>0.99
Avelumab (PD-L1)	0 (0.0%)	1 (10.0%)	1 (4.2%)	0.42
Durvalumab (PD-L1)	1 (7.1%)	0 (0.0%)	1 (4.2%)	>0.99
Other	0 (0.0%)	0 (0.0%)	0 (0.0%)	-
Cemiplimab (PD-1)	0 (0.0%)	0 (0.0%)	0 (0.0%)	-
ICI’s ever received before first AKI episode, n (%)				
Ipilimumab (CTLA-4)	2 (14.3%)	3 (30.0%)	5 (20.8%)	0.62
Tremelimumab (CTLA-4)	0 (0.0%)	0 (0.0%)	0 (0.0%)	-
Nivolumab (PD-1)	2 (14.3%)	4 (40.0%)	6 (25.0%)	0.19
Pembrolizumab (PD-1)	10 (71.4%)	4 (40.0%)	14 (58.3%)	0.21
Atezolizumab (PD-L1)	2 (14.3%)	1 (10.0%)	3 (12.5%)	>0.99
Avelumab (PD-L1)	0 (0.0%)	1 (10.0%)	1 (4.2%)	0.42
Durvalumab (PD-L1)	1 (7.1%)	0 (0.0%)	1 (4.2%)	>0.99
Other	0 (0.0%)	0 (0.0%)	0 (0.0%)	-
Cemiplimab (PD-1)	0 (0.0%)	0 (0.0%)	0 (0.0%)	-
Any TIN drugs, n (%)	8 (57.1%)	1 (10.0%)	9 (37.5%)	0.019^a^
Antibiotics	2 (14.3%)	0 (0.0%)	2 (8.3%)	0.49
NSAIDS	2 (14.3%)	0 (0.0%)	2 (8.3%)	0.49
Proton pump inhibitors	5 (35.7%)	1 (10.0%)	6 (25.0%)	0.34
Cisplatin, n(%)	3 (21.4%)	1 (10.0%)	4 (16.7%)	0.62
TKI, n(%)	1 (7.1%)	3 (30.0%)	4 (16.7%)	0.27
IRAE before AKI, n(%)	6 (42.9%)	5 (50.0%)	11 (45.8%)	>0.99
Rash	2 (14.3%)	1 (10.0%)	3 (12.5%)	>0.99
Colitis	1 (7.1%)	0 (0.0%)	1 (4.2%)	>0.99
Hepatitis	1 (7.1%)	0 (0.0%)	1 (4.2%)	>0.99
Pneumonitis	1 (7.1%)	1 (10.0%)	2 (8.3%)	>0.99
Thyroid disease	1 (7.1%)	2 (20.0%)	3 (12.5%)	0.55
Hypophysitis	0 (0.0%)	0 (0.0%)	0 (0.0%)	-
Primary adrenal insufficiency	0 (0.0%)	0 (0.0%)	0 (0.0%)	-
Type 1 DM	0 (0.0%)	0 (0.0%)	0 (0.0%)	-
Myocarditis	0 (0.0%)	0 (0.0%)	0 (0.0%)	-
Other	4 (28.6%)	2 (20.0%)	6 (25.0%)	>0.99
IRAE at time of AKI, n(%)	7 (50.0%)	2 (20.0%)	9 (37.5%)	0.21
Rash	3 (21.4%)	0 (0.0%)	3 (12.5%)	0.24
Colitis	1 (7.1%)	0 (0.0%)	1 (4.2%)	>0.99
Hepatitis	0 (0.0%)	0 (0.0%)	0 (0.0%)	-
Pneumonitis	1 (7.1%)	1 (10.0%)	2 (8.3%)	>0.99
Thyroid disease	1 (7.1%)	0 (0.0%)	1 (4.2%)	>0.99
Hypophysitis	0 (0.0%)	0 (0.0%)	0 (0.0%)	-
Primary adrenal insufficiency	0 (0.0%)	0 (0.0%)	0 (0.0%)	-
Type 1 DM	0 (0.0%)	0 (0.0%)	0 (0.0%)	-
Myocarditis	1 (7.1%)	0 (0.0%)	1 (4.2%)	>0.99
Other	1 (7.1%)	1 (10.0%)	2 (8.3%)	>0.99
Renal recovery, n (%)	7 (50.0%)	1 (10.0%)	8 (33.3%)	0.079
CRP (mg/l) at time of AKI, median (IQR)	14.3 (5.0, 31.3)	5.8 (3.0, 8.2)	7.5 (4.1, 27.6)	0.28
uRBP/Cr at time of AKI, median (IQR)	2208 (918, 16,067)	1530 (86, 26,735)	1783 (673, 20,588)	0.83
AKI stage, n (%)				0.25
1	9 (64.3%)	6 (60.0%)	15 (62.5%)	
2	4 (28.6%)	1 (10.0%)	5 (20.8%)	
3	1 (7.1%)	3 (30.0%)	4 (16.7%)	

AKI, acute kidney injury; CKD, chronic kidney disease; COPD, chronic obstructive pulmonary disease; Cr, creatinine; CRP, C-reactive protein; CTLA-4, cytotoxic T lymphocyte–associated antigen 4; DM, diabetes mellitus; HTN, hypertension; IBD, inflammatory bowel disease; ICI, immune checkpoint inhibitors; IQR, interquartile range; NSADS, nonsteroidal anti-inflammatory drugs; PD-1, programmed cell death 1; PD-L1, programmed death ligand 1; TIN, tubulo-interstitial nephritis; TKI, tyrosine kinase inhibitor; uRBP/Cr, urine retinol binding protein-to-creatinine ratio.

a *P*-values in bold denote statistical significance at the 0.05 α-level.Unless otherwise indicated, timing is at initiation of ICI therapy.*P*-values are derived from the equal variance *t*-test for normally distributed variables, the Wilcoxon rank sum test for non-normally distributed variables, the χ^2^ test for categorical variables where the expected call counts were ≥5, and the Fisher exact test for categorical variables with an expected cell count of <5. *P*-values in bold denote statistical significance at the 0.05 α-level.

Kidney biopsy was performed in 10 of the 14 (71%) AKI-ICI patients and 4 of the 10 (40%) patients with AKI-other. Among these, AIN was the predominant acute lesion in the AKI-ICI group (*n* = 10, 100%), whereas in the AKI-other group, acute lesions were acute tubular injury in 2 (50%) patients and renal thrombotic microangiopathy in 2 (50%) patients, with mild to moderate interstitial fibrosis or tubular atrophy in all 4 (100%) patients. In the AKI-ICI patient group, 9 (90%) had acute tubular injury and 2 (20%) had manifest tissue eosinophilia. Histologic features of these patients are presented in Supplementary Table S6 for the AKI groups and Supplementary Table 12 for all groups.

### Urine and Plasma Cytokines

**Figure 2:**
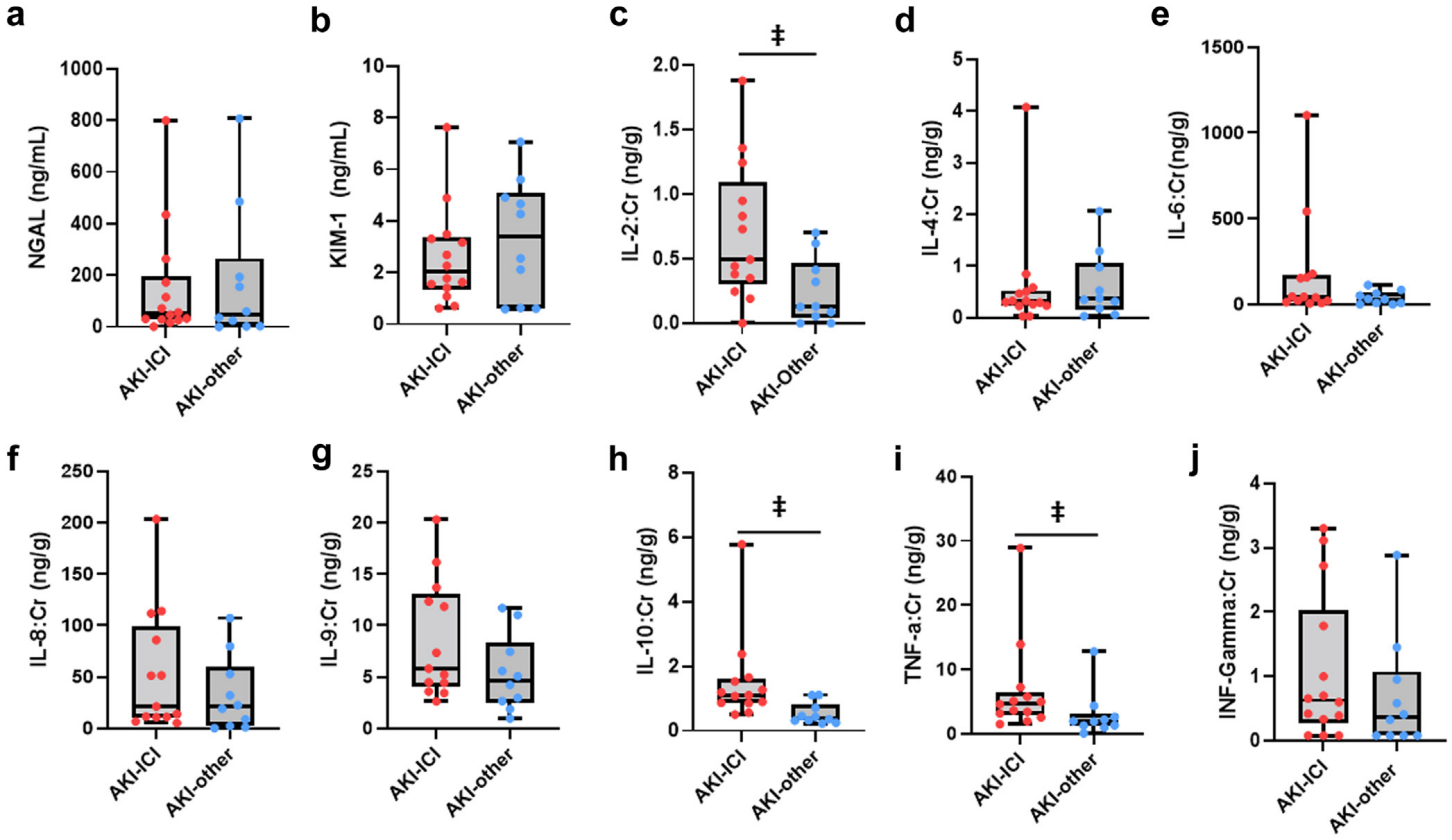
Urine markers of kidney injury and cytokines. Box and whisker plots of markers of kidney and tubular injury in AKI-ICI vs. AKI-other groups: (a) NGAL, (b) KIM-1, (c) IL-2, (d) IL-4, (e) IL-6, (f) IL-8, (g) IL-9, (h) IL-10, (i) TNF-α, (j) INF-γ levels in the urine. The boxes extend from the 25th percentile to the 75th percentile and are bisected by the median; the whiskers extend to the minimum and maximum value. *P*-values derived from Wilcoxon rank sum test. **ǂ =***P* < 0.05. AKI, acute kidney injury; ICI, immune checkpoint inhibitors; IL, interleukin; KIM, kidney injury molecule; NGAL, neutrophil gelatinase-associated lipocalin.

We evaluated various inflammatory and tubular cytokines concomitantly in the urine and plasma. To characterize injury in patients with AKI, we also evaluated levels of urine neutrophil gelatinase-associated lipocalin and kidney injury molecule-1 **(**see Table 3 and Figure 2). We found that urine IL-2, IL-10, and TNF-α were all significantly elevated in AKI-ICI patients compared to AKI-other patients (Figure 2). The discriminatory ability of TNF-α on AKI cause was particularly strong (area under the curve = 0.814, 95% confidence interval: 0.623−1.000), see Supplementary Figure S1. Correlation analyses performed among the urine biomarkers are presented in Supplementary Table S7. IFN-γ was significantly positively associated with IL-4 and IL-8, IL-6 was positively associated with IL-8, and TNF-α was positively associated with both IL-8 and IL-10. When compared to the healthy control group (see Supplementary Table S13), only urine IL-10 and TNF-α were consistently elevated in the AKI-ICI group compared to both controls and AKI-others.

**Table 3:** Urine cytokines, by AKI cause

Urine cytokine	AKI-ICI (*N* = 14)	AKI-Other (*N* = 10)	*P*-value
IFN (ng/g)	0.63 (0.35, 1.59)	0.37 (0.08, 0.86)	0.26
IL-2 (ng/g)	0.49 (0.35, 0.95)	0.13 (0.06, 0.39)	0.020^a^
IL-4 (ng/g)	0.31 (0.24, 0.50)	0.36 (0.21, 0.87)	0.56
IL-6 (ng/g)	44.8 (20.5, 158)	30.2 (7.80, 60.9)	0.22
IL-8 (ng/g)	21.3 (11.1, 85.8)	20.9 (3.96, 47.8)	0.35
IL-9 (ng/g)	6.59 (4.44, 13.4)	4.65 (2.74, 6.99)	0.079
IL-10 (ng/g)	1.11 (0.87, 1.53)	0.39 (0.32, 0.65)	0.002^a^
TNF-α (ng/g)	4.80 (3.23, 6.89)	1.95 (1.29, 2.51)	0.010^a^
NGAL (ng/ml)	51.7 (29.8, 159)	48.1 (8.21, 185)	0.82
KIM (ng/ml)	2.02 (1.45, 3.28)	3.41 (1.00, 4.85)	0.60

AKI, acute kidney injury; ICI, immune checkpoint inhibitors; IFN, interferon; IL, interleukin; KIM, kidney injury molecule; NGAL, neutrophil gelatinase-associated lipocalin; TNF, tumor necrosis factor.

*P*-values are derived from the Wilcoxon rank sum test.

a *P*-values in bold denote statistical significance at the 0.05 α-level.Summary statistics reported are median (IQR).

To evaluate whether urine biomarkers were being produced in the kidneys or filtered from the blood, we also measured plasma cytokines. However, no significant differences were observed between AKI groups. (see Supplementary Table S8). When compared to controls, only Plasma IL-6 and IL-10 were found to be significant in the overall analysis of variance test (see Supplementary Table S14).

### TNF-α Expression in Kidney Tissue

We also examined TNF-α expression in kidney tissues from the 2 AKI groups AKI-ICI (*n* = 4) and AKI-other (*n* = 4), as well as in kidney tissues from the healthy controls (*n* = 3) **(**see Supplementary Figure S2). Significant differences were only observed between the AKI-ICI and healthy control groups. Correlation analyses comparing urine TNF-α and TNF-α tissue expression also showed a positive association (Spearman’s rank correlation = 0.73, *P* = 0.009).

### Immune Cell Phenotype Characterization in PBMCs in Patients With AKI Status

We characterized immune cell phenotypes in peripheral blood of the patients who developed AKI while on ICI therapy using mass cytometry methods. This high parameter cytometry system allowed us to simultaneously quantify multiple immune cell populations with single-cell resolution. Because certain drugs are expected to have a significant impact on the peripheral blood immune profile, patients using immunosuppressive drugs at the time of AKI were excluded from this analysis. In the subset of patients with available PBMCs and who were not on any immunosuppressive at the time of their AKI event, we found no significant differences in the single cell phenotype by cell assignment, although there were elevated counts of natural killer T cells in the AKI-ICI group compared to those with AKI-other (see Supplementary Table S9).

**Figure 3:**
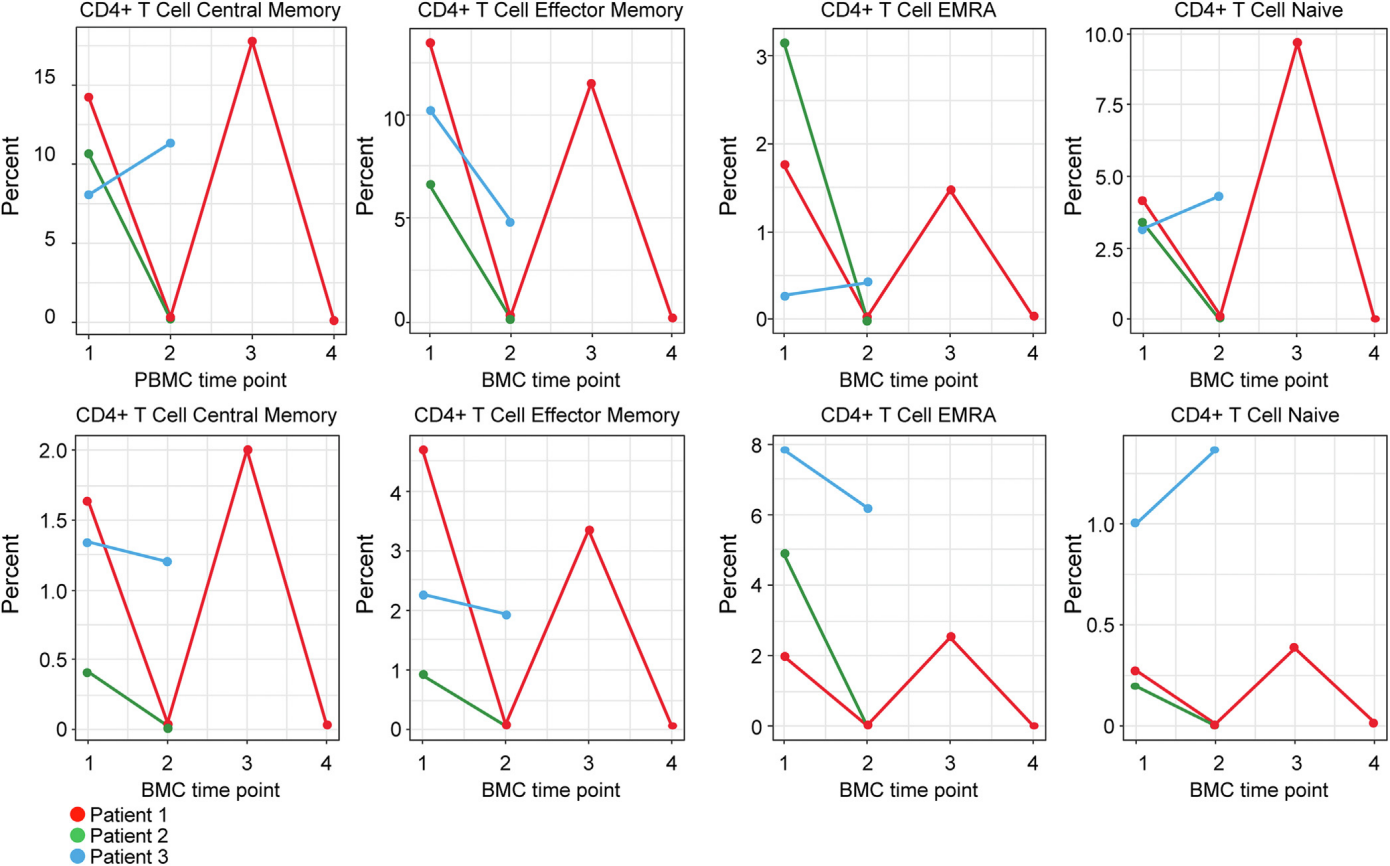
Evaluation of immunologic profile of 3 patients during AKI-ICI. PBMC time point 1: immune cell subsets at time of AKI-ICI without immunosuppression. PBMC time point 2: immune cell subsets during tapering of corticosteroids. PBMC time point 3: elevation of immune cells subsets during rechallenge, only for patient #1 (Red). PBMC time point 4: overall decrease of immune cells subsets of patient# 1 (Red) after resuming corticosteroids during rechallenge. AKI, acute kidney injury; ICI, immune checkpoint inhibitor; PBMC, peripheral blood monocytes cell.

Three patients in the AKI-ICI group also had subsequent PBMCs collected following AKI while tapering immunosuppressive therapy for the treatment of interstitial nephritis, as illustrated in Figure 3. We observed a downtrend of most of the immune cells subsets while on corticosteroid taper (time point 2). Patient #1 (red) also had an additional 2 subsequent PBMC time point collections. Time point 3 demonstrates reconstitution of immune cell population during rechallenge with ICI therapy without corticosteroids, whereas during time point 4, a decrease of immune cells subsets is observed when corticosteroids are resumed again. These 2 time points (time points 3 and 4) were also associated with concurrent elevation and subsequent decrease of creatinine, respectively (data not shown).

### Spatial Immune Cell Phenotype Characterization in Kidney Tissue From Imaging Mass Cytometry Data

**Figure 4:**
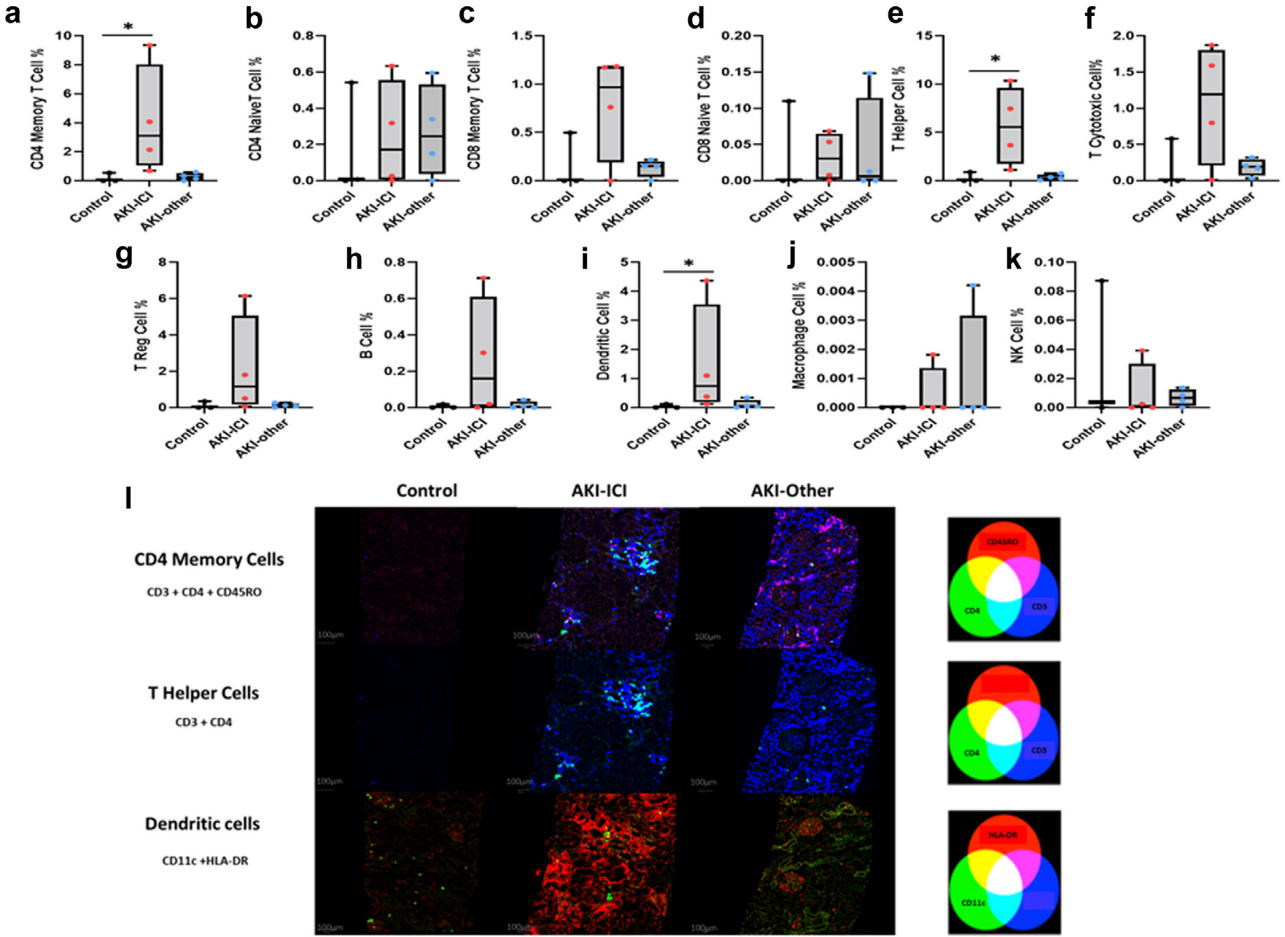
Differences in immune cell phenotype in the kidney tissue between AKI-ICI, AKI-other and healthy control patients. Box and whisker plots of immune cell subtypes (colocalization as described in methods section) in AKI-ICI vs. AKI-other and control groups of (a) CD4 memory T cells, (b) CD4 naïve T cells, (c) CD8 memory T cells, (d) CD8 naïve T cells, (e) T helper cells, (f) Cytotoxic T cells, (g) T reg cells, (h) B cells, (i) Dendritic cells, (j) Macrophage cells, (k) NK cells. The boxes extend from the 25th percentile to the 75th percentile and are bisected by the median; the whiskers extend to the minimum and maximum value. *P*-values derived from the Kruskal-Wallis test. For *post hoc* tests, Dunn’s multiple comparison test was used, with *P*-values adjusted with the Holm method. AKI, acute kidney injury; ICI, immune checkpoint inhibitors. (l) Representative images of immune cells subsets of CD4 memory, T helper and dendritic cells in the spatial context of the renal cortex biopsy: CD4 Memory T cells: Cell classifications show CD4 single positive (green), CD45RO single positive (red), and CD3 single positive (blue) cells. Double and triple positive cells are colored according to the additive color mixing model shown above. T Helper Cells: Cell classifications show CD4 single positive (green), CD3 single positive (blue), and CD3/CD4 double positive helper cells (cyan). Dendritic Cells: Cell classifications show CD11c single positive (green), HLA-DR single positive (red), and CD11c/HLA-DR double positive dendritic cells (yellow). HLA-DR expression is predominantly along the renal tubular epithelial cells in the renal cortex. AKI, acute kidney injury; ICI, immune checkpoint inhibitors. Immune cells are defined as per markers listed in Supplementary Table S5 for each cell phenotype. Cell percentages listed are as a percentage of all cells detected. ∗ = *P* < 0.05. AKI-ICI vs. control. AKI, acute kidney injury; ICI, immune checkpoint inhibitors.

We used imaging mass cytometry to evaluate the relative abundance of individual immune cell subsets within the kidney immune microenvironment in a subset of the AKI and healthy donor patients with available kidney biopsies (see Figure 4a–k). In general, control kidney tissues (as expected) demonstrated low abundance of all immune cell subtypes observed; whereas AKI-ICI demonstrated elevated immune cell abundance when measured as a percentage of the total cells observed within each ROI. Specifically, when evaluated on a percent positive score basis, the presence of CD4 memory T cells, T helper cells, and dendritic cells were significantly elevated in the AKI-ICI group in comparison to the control tissues (*P* < 0.05 for all), indicating a potential role of these immune cell subsets in AKI-ICI (see Table 4). When comparing these colocalized immune cells groupings among patients with AKI only, CD4 memory, T helper and dendritic cell levels were significantly elevated in patients with AKI-ICI compared to AKI-other (*P* < 0.05 for both, Table 5). The overlap of these immune cell populations, positive cells identified for each immune cell subtype were assigned red, green, or blue colors, and visualized in Figure 4l.

**Table 4:** Immune cell phenotype subgroups in kidney biopsies, by AKI cause (including healthy controls)

Cell %	AKI-ICI (*N* = 4)	AKI-Other (*N* = 4)	Controls (*N* = 3)	*P*-value
CD4 memory T cells	3.10 (1.77, 5.39)	0.25 (0.11, 0.40)	0.01 (0.00, 0.27)	0.028^a^
CD4 naïve T cells	0.17 (0.02, 0.40)	0.01 (0.01, 0.01)	0.00 (0.00, 0.02)	0.34
CD8 memory T cells	0.97 (0.57, 1.18)	0.15 (0.11, 0.17)	0.00 (0.00, 0.25)	0.26
CD8 naïve T cells	0.03 (0.01, 0.06)	0.01 (0.00, 0.05)	0.00 (0.00, 0.05)	0.89
T helper subset	5.57 (3.04, 8.18)	0.30 (0.18, 0.46)	0.02 (0.02, 0.46)	0.027^a^
T cytotoxic subset	1.19 (0.60, 1.66)	0.19 (0.14, 0.24)	0.00 (0.00, 0.29)	0.18
T reg cells	1.16 (0.40, 2.89)	0.10 (0.06, 0.16)	0.00 (0.00, 0.17)	0.14
B cells	0.16 (0.01, 0.40)	0.00 (0.00, 0.02)	0.00 (0.00, 0.01)	0.27
Dendritic cells	0.74 (0.31, 1.92)	0.04 (0.03, 0.12)	0.00 (0.00, 0.06)	0.035^a^
Macrophages	0.00 (0.00, 0.00)	0.00 (0.00, 0.00)	0.00 (0.00, 0.00)	0.65
NK cells	0.00 (0.00, 0.01)	0.01 (0.00, 0.01)	0.00 (0.00, 0.05)	0.74

AKI, acute kidney injury; ICI, immune checkpoint inhibitors; NK cells, natural kidney cells.

a Statistical significance at the 0.05 α-level. Significant for groups AKI-ICI vs. controls in *post hoc* test adjused for multiple comparisons. Summary statistics reported are median (IQR). *P*-values are derived from Kruskal-Wallis tests. For *post hoc* tests, Dunn’s multiple comparison test was used, with *P*-values adjusted with the Holm method.

**Table 5:** Immune cell phenotype subgroups in kidney biopsies, by AKI cause

Cell %	AKI-ICI (*n* = 4)	AKI-Other (*n* = 4)	*P*-value
CD4 memory T cells	3.10 (1.77, 5.39)	0.25 (0.11, 0.40)	0.021^a^
CD4 naïve T cells	0.17 (0.02, 0.40)	0.01 (0.01, 0.01)	0.19
CD8 memory T cells	0.97 (0.57, 1.18)	0.15 (0.11, 0.17)	0.25
CD8 naïve T cells	0.03 (0.01, 0.06)	0.01 (0.00, 0.05)	0.77
T helper subset	5.57 (3.04, 8.18)	0.30 (0.18, 0.46)	0.021^a^
T cytotoxic subset	1.19 (0.60, 1.66)	0.19 (0.14, 0.24)	0.25
T reg cells	1.16 (0.40, 2.89)	0.10 (0.06, 0.16)	0.15
B cells	0.16 (0.01, 0.40)	0.00 (0.00, 0.02)	0.24
Dendritic cells	0.74 (0.31, 1.92)	0.04 (0.03, 0.12)	0.043^a^
Macrophages	0.00 (0.00, 0.00)	0.00 (0.00, 0.00)	0.85
NK cells	0.00 (0.00, 0.01)	0.01 (0.00, 0.01)	0.55

AKI, acute kidney injury; ICI, immune checkpoint inhibitor; NK cells, natural killer cells.

Summary statistics reported are median (IQR). *P*-values are derived from the Wilcoxon rank sum test.

a Statistical significance at the 0.05 α-level.

Similar results were observed when we evaluated the relative abundance of single marker positive cells across the 2 AKI groups (see Supplementary Table S10), with significant differences observed between groups for the markers CD4, CD11c, CD68, and CD45. In addition, human leukocyte antigen-DR+ cells were also present in higher abundance in the AKI-ICI group, but the differences observed did not reach statistically significance (*P* > 0.05).

**Figure 5:**
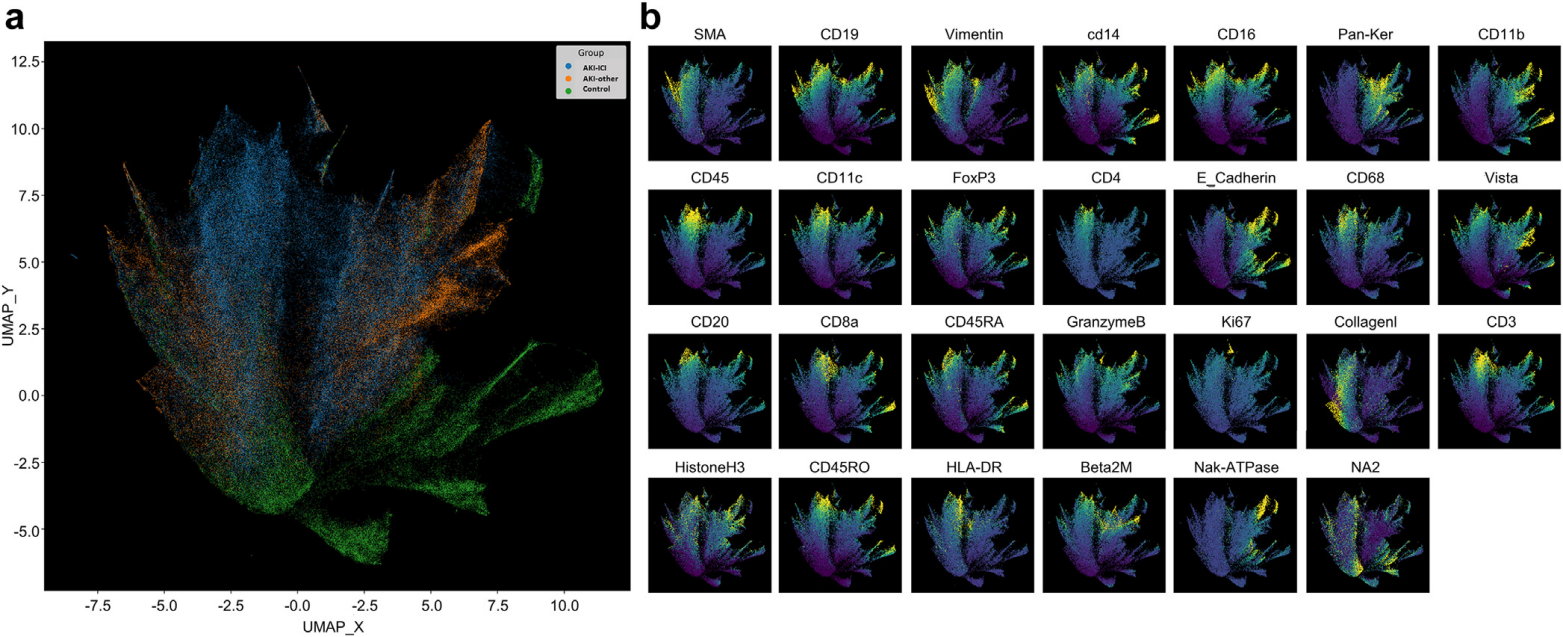
Unsupervised UMAP 2D projection of imaging mass cytometry data. (a) colored by different patient groups: uniform manifold approximation and projection (UMAP) for cells from AKI-ICI (blue), ICI-Other (orange), and Control (green) patients. Per-cell statistics were pooled for all 3 patient groupings, and UMAP was computed using the mean cell intensity for each of the 27 markers. Clustering approaches were not used, and rather rely on the underlying label of which patient grouping the cell was derived from (as described in the methods section). UMAP_X and UMAP_Y represent arbitrary dimensionless axes for projecting the dimensionality-reduced cells. Cells in close proximity indicate phenotypic similarity as defined by marker expression patterns, whereas distal cells are presumed to have a greater dissimilarity. Visual interpretation indicates a high extent of homogeneity of AKI-other cells with AKI-ICI, more than ICI-Other with control. (b) Panel of UMAP projection images for the 26 markers and DNA intercalator (NA2) utilized in this study. Color scale range (blue to yellow) corresponds to least to maximum marker intensity. UMAP_X and UMAP_Y axes match with Figure 5A to allow for direct comparison between marker intensity and patient grouping. HLA-DR, human leukocyte antigen; SMA, spinal muscular atrophy.

An alternative, unsupervised method to evaluate differences between patient groups is to use dimensionality reduction to group cells exhibiting similar characteristics together to investigate the relative relationships between individual antibody markers and the patient groups themselves. To accomplish this, we performed uniform manifold approximation and projection (UMAP) projection to reduce 26 antibodies (plus one DNA intercalator) down to a 2-dimensional projection, in which cells with similar marker characteristics likely corresponding to similar phenotypes cluster together. The UMAP projection shown on Figure 5a is color coded by patient group, indicating that there is some segregation between the patient groups, which reveal themselves as a greater preponderance of one particular patient group color within specific regions of the UMAP projection. The relative intensity of each biomarker within the same UMAP projection, in which low antibody staining is shown in blue and high antibody staining is shown in yellow is presented in Figure 5b. By comparing the intensity information in Figure 5b to Figure 5a, we observed that high intensity CD4, CD11c, CD68, and CD45 cells do localize preferentially to regions where AKI-ICI patients are segregated, which corroborates our findings made using percent positive analysis. In contrast, regions where control patient samples are segregated tend to have low intensities of most of the immune markers observed, whereas AKI-other regions show intermediate intensities for many of the markers, with some evidence of increased signal in CD11b, FoxP3, CD14, and CD16, also matching some of the differences observed in marker abundance in Supplementary Table S15. The fact that we observed similar relationships between patient groups and immune marker abundance helps to validate and strengthen the conclusions drawn from both the supervised percent positive data analysis and the unsupervised UMAP projection and marker intensity relationships.

## Discussion

In this pilot study, we have demonstrated that urine TNF-α, IL-2, and IL-10 levels are higher in patients with AKI-ICI compared to those with AKI-other. These findings in conjunction with strong expression of TNF-α in the kidney biopsies suggest that TNF-α originates primarily in the kidneys. Therefore, our results suggest that urinary levels of TNF-α are higher in patients with AKI-ICI with pathology demonstrating AIN, and thus may serve as a useful biomarker to distinguish AKI-ICI from other clinical causes of AKI while on ICI therapy. The results of this study also corroborate the findings of Moledina *et al.*,^12^ who have previously shown that urinary TNF-α improves discrimination over clinicians’ prebiopsy diagnosis in patients with AIN. However, in our study, despite urine IL-9 levels being elevated in patients with AKI-ICI, we did not find statistically significant differences compared to AKI-other patients.

Another important and novel finding from this study is the kidney tissue immunoprofiling characterization in patients with AKI-ICI. Using imaging mass cytometry, we found an abundance of specific immune cells including CD4 memory, T helper, and dendritic cells in the kidney tissue of patients who developed AKI-ICI. These findings suggest that dendritic cell-T cell interaction within the AKI-ICI and the subsequent T cell activation may contribute to the underlying pathophysiology. Interestingly, a recent study published by Lozano *et al.*^23^ also discovered that CD4 memory T cells in abundance in the peripheral blood was associated with severe irAEs independently of type of organ system involved using mass cytometry by time of flight, which further corroborates our findings.

Previous studies have detected both CD4+ and CD8+ T cells in kidney tissue of patients with AKI-ICI^24^^,^^25^ and this has also been reported in cases of patients with AIN.^26^ In the current study, whereas more CD8+ T cells were found in the kidney tissue of patients with AKI-ICI than AKI-other, these differences did not reach statistical significance. CD4+ T cells help CD8+ T cells to maximize CD8+ T cell population expansion during a primary immune response facilitating the generation of memory CD8+ T cell populations.^27^ Similar to our findings of the infiltrating CD4+ T cells, the main CD8+ subset in the AKI-ICI kidneys were the memory T cells. Given the elevated levels of proinflammatory cytokines detected in the urine of AKI-ICI patients, specifically TNF-α, this suggests that ICI-induced inflammation is contributing to the patient’s kidney injury. IL-10 was also elevated systemically and in the urine, which is not unexpected, because IL-10 has a role in limiting host immune responses and regulating inflammation and autoimmune states, even in patient with irAES.^28^^,^^29^ TNF-α is a pleiotropic cytokine that can mediate the inflammatory response, regulate immune function by promoting immune cells activation and recruitment, and may trigger cell proliferation, differentiation, and apoptosis.^30^ TNF-α is also known to increase the expression of HLA- molecule, thereby inducing activation of antigen- presenting cells.^31^ This might explain the trend in our study of increased expression of HLA-DR in the kidney tissue of those with AKI-ICI. Notably, the dendritic cell numbers were also found to be abundant in the AKI-ICI kidneys. Taken together, these findings suggest that ICI-induced AIN could be caused by the loss of peripheral tolerance of autoreactive T cells against tubular cells.^25^^,^^32^ Immunotherapy reduces normal tolerance to medications associated with AIN, which may be responsible for the greater incidence of kidney irAEs in patients on ICIs and proton pump inhibitors or nonsteroidal inflammatory drugs, for example. This theory is supported by recent studies showing the association between biopsy-proven AIN in ICI-treated patients and previous exposure to AIN-associated drugs.^4^^,^^5^^,^^8^

In this study, we did not find significant differences in the single cell phenotype by cell assignment of PBMCs; however, natural killer T cells tended to be elevated in the AKI-ICI group compared to those with AKI-other. Furthermore, from the assessment of detailed immunophenotyping of peripheral blood from 3 patients who developed AKI-ICI and were subsequently controlled with corticosteroids, we observed changes in the immune cell response. We also discovered that when further stratifying T cells into CD4+ and CD8+ subsets, most patients had these immune cells decreased with administration of corticosteroids. Notably, for patient #1 we noticed an increase in the proportions of circulating CD8+ T cells and CD4+ T cells after rechallenging, and this was accompanied by concomitant elevations of creatinine. This phenomenon was also identified by Lozano *et al.* showing early T cell clonal expansion at the timing of severe irAEs.^23^ In our patient #1, this T cell clonal expansion was then reversed by resuming corticosteroids. These findings demonstrate that ICIs can lead to widespread T cell activation during the time of AKI-ICI, followed by successful control during immunosuppression. Therefore, T cell monitoring may help in guiding immunosuppressive therapy in patients with AKI-ICI, including optimal steroid dosing (tapering vs. increase).

### Strengths and Limitations

To our knowledge, this is the first study to describe the use of novel biomarkers and immune cells subsets that are important regulators of kidney-associated irAEs leading to AKI-ICI. We have shown that TNF-α is a pivotal cytokine contributing to AKI-ICI; this is an important finding, becuase TNF-α blockade could have steroid-sparing potential in achieving durable and complete renal recovery in patients with refractory AKI-ICI as previously shown by Lin *et al.*^33^ Moreover, this is the first study to provide a detailed overview at a single cell level of the immune microenvironment from kidney biopsies in patients who developed AKI while on ICI therapy. This was possible using advanced multiplexed imaging technology and multiparametric analysis obtained from a single formalin-fixed paraffin-embedded tissue section. Combining the ability to stain with highly quantitative multiplexed mass cytometry antibody panels with advanced capabilities to identify the spatial localization of single cells within a kidney biopsy image and phenotype those cells using both supervised thresholding and unsupervised dimensionality reduction methods is of paramount importance. This can help to resolve differences in abundance of specific immune cell types that may be contributing to AKI-ICI mediated kidney damage while preserving kidney compartmental structure.

However, our study does have several limitations. Our patient cohort reflects a Midwestern, largely Caucasian population. Therefore, we cannot generalize these findings to other patient populations. Because of our limited sample size, our study did not allow for multivariable adjusted analyses to control for potential confounders. Furthermore, we did not have a control group of ICI- treated patients without AKI, given that kidney biopsies would not be clinically indicated in this population. Larger cohort studies are needed to develop multivariable risk prediction models for incident AKI-ICI. Because this was an exploratory study, results should be considered as preliminary and subject to more rigorous testing and replications in future studies.

### Conclusions

Taken together, our study suggests that strong TNF-α upregulation in conjunction with an increase of specific immune cell subsets in the kidney tissue are associated with AKI-ICI and may serve as both biomarkers and potential targets for therapeutic intervention for AIN. Furthermore, immune cell profiling during ICI therapy may help to guide management of AKI-ICI. Further multiomics studies on cytokine levels and immune cell profiling in both peripheral blood and kidney tissue in a larger cohort will help to validate the initial findings seen here, as well as provide novel biologic insights into the pathobiological mechanisms of AKI-ICI, with the ultimate goal of improving patient outcomes.

## Disclosure

NL has stocks in Checkpoint Therapeutics and is on the advisory board of AbbVie, Takeda, and Aduro. TM is an employee of Deciphex Inc. SM has received royalties from Sorrento Pharma. All other authors have no competing interests.

## Supplementary Material

**Supplementary Methods.**

**Figure S1.** Receiver operating curve (ROC) for prediction of AKI type.

**Figure S2.** TNF-α expression in kidney tissue.

**Table S1.** Criteria AKI-ICI and AKI-other cohorts.

**Table S2.** Human enhanced PBMC phenotyping panel for mass cytometry analysis.

**Table S3.** Detection range of the assays.

**Table S4.** Human imaging mass cytometry panel.

**Table S5.** Immune cell definitions.

**Table S6.** Histologic features of *n* = 14 biopsied patients, by cause of AKI.

**Table S7.** Correlation between urine cytokines.

**Table S8.** Plasma cytokines by AKI status.

**Table S9.** Immune cell phenotype in peripheral blood.

**Table S10.** Immuno cell phenotype in kidney tissue, by AKI status (single cells).

**Table S11.** Demographic and clinical characteristics, by AKI status and control group.

**Table S12.** Histologic features of biopsied patients in the AKI-ICI and AKI-other and control group.

**Table S13.** Urine cytokines, by AKI status and control group.

**Table S14.** Plasma cytokines by AKI status and control group.

**Table S15.** Immuno cell phenotype in kidney tissue by AKI status and control group (by single cells markers).

**STROBE Statement.**
