# Supplementary material for: Cytokines and Immune Cell Phenotype in Acute Kidney Injury Associated With Immune Checkpoint Inhibitors
Source: Kidney Int Rep. 2022 Dec 5;8(3):628–41. doi: 10.1016/j.ekir.2022.11.020 (PMC10014345; doi:10.1016/j.ekir.2022.11.020)
Supplement: Supplementary File (PDF) [file mmc1.docx]

Supplementary Appendix

Table of Contents

**Supplementary Methods**………………………………………………...…………………………………………………....….**2-5**

**Supplemental Tables6-15**

Table S1. Criteria AKI-ICI and AKI-other cohort………………………...……………………………….......................................**6**

Table S2. Human Enhanced PBMC Phenotyping Panel for Mass Cytometry Analysis ………........................................................**7**

Table S3. Human Imaging Mass Cytometry Panel.……………………….…………………….……………………………..........**9**

Table S4. Immune cell definitions: ……………….………...….………..........................................................................................**10**

Table S5. Cytokine detection range assays……………………………………………………………………………………….….8

Table S6. Histologic features of biopsied patients by cause of AKI ………………........................................................................**11**

Table S7. Correlation between Urine Cytokines………….………..................................................................................................**12**

Table S8. Plasma cytokines by AKI status …………………………………….………………………………….……….……....**13**

Table S9. Immune cell Phenotype in Peripheral Blood …………………………………….…………………………….…….….**14**

Table S10. Immuno Cell Phenotype in Kidney Tissue (single cells) …………………………………………………….…….….**15**

**Supplemental Figures**……………………….………………………………………………….……………..........................**16-17**

Figure S1. Receiver operating curve (ROC) for prediction of AKI type ………….........................................................................**16**

Figure S2. TNF-α expression in kidney tissue ……………………………………………………………………...….….…...….**17**

**Tables from 3-group comparison**…………………………...……………………………….………....……………….……**18-22**

Table S11. Demographic and clinical characteristics, by AKI status and control group……...……………….…….**18**

Table 12. Histologic features of biopsied patients in the AKI-ICI and AKI-other and control group……….………**19**

Table 13. Urine cytokines, by AKI status and control group…………………………………….………….……….**20**

Table 14. Plasma cytokines by AKI status and control group…………………………………………………….….**21**

Table 15. Immuno Cell Phenotype in Kidney Tissue (single cells) by AKI status and control group...…….……….**22**

**References**....…………………………………………………………………………………………………………**23**

**Supplementary Methods**

Imaging Mass Cytometry Staining of Kidney Biopsies

All tissue staining and slide preparation was performed by the Mayo Clinic Pathology Research Core. Formalin fixed paraffin embedded tissue sections derived from renal blocks of patients with AKI-ICI, AKI-other and controls were stained with our full antibody panel (**Supplemental Table S4**). Briefly, slides were baked for 60 minutes in a 60°C oven and then cooled for 5 minutes before loading on to a Bond RX autostainer (Leica) for automated slide preparation prior to staining. Slides were deparaffinized with xylol and rehydrated through a graded alcohol series and were blocked with Superblock solution (ThermoFisher) before a final wash with PBS supplemented with 0.05% Tween and 1% BSA (PBS-TB). Slides were manually stained overnight in a humidity chamber at 4°C with a cocktail of the antibodies diluted to working concentration. On the following day, slides were washed with PBS-TB and then stained with an iridium containing nucleic acid intercalator (Fluidigm) to label cell nuclei. Slides were washed with PBS-TB twice before a final wash with water and drying for 20 minutes at room temperature.^1^

Imaging Mass Cytometry Data Acquisition

Multiple regions of interest (ROI), ranging from 4 to 8 ROIs per biopsy, were annotated on H&E images using Aperio ImageScope software (Leica Biosystems). Those ROIs, measuring from 1 to 2 mm2 in area, were then selected for laser ablation, which was performed on a Helios time-of-flight mass cytometer (CyTOF) connected to a Hyperion Imaging System (Fluidigm). Laser ablation using the Hyperion software (version 7.0.5189.0) occurred over 3 consecutive days, at a resolution of 1 micron and frequency of 200 Hz. To ensure highly quantitative results for each isotope, the performance stability of the system was calibrated using a 3-element full coverage tuning slide embedded with 3 metal elements spanning the isotope detection range, 89Y, 140Ce and 175 Lu (Fluidigm). Metals selected for antibody conjugation and staining were optimized to spread isotope masses across the mass range of the time-of-flight detector, and the antibody cocktail panel concentrations adjusted to ensure robust staining for each marker selected within the panel. Representative images from the renal cortex sections were generated using the MCD Viewer software (version 1.0.560.2; Fluidigm). Additional formatting and layout of these images was performed using Adobe Photoshop (version 19.1.3).

Imaging Mass Cytometry (IMC) Tissue and Cellular Segmentation Image Analysis Methods

Digital image analysis of IMC data was performed in QuPath version 0.3.2. Prior to any analysis, quality control was performed by visually evaluating representative images and channels in consultation with a pathologist. A reliable pre-trained deep learning algorithm, StarDist^2^, was used to perform cell segmentation, utilizing the DNA intercalator channel to isolate and separate individual nuclei, with cytoplasm simulated around each nucleus. The ROI images (67 ROIs across 3 treatment groups) were loaded into one QuPath project, and individual 500x500 um representative areas were selected within ROIs from each case in order to generate a single training image to use for subsequent parameter optimization. Several cell segmentation methods were attempted on the data, however the relatively low resolution (1 µm/pixel) of IMC images posed challenges to conventional watershed segmentation methods. Here, we used Universal StarDist for QuPath (https://github.com/MarkZaidi/Universal-StarDist-for-QuPath) to apply the publicly available pretrained StarDist deep learning based models on our IMC data.^3^ Minimal hyperparameter optimization was required, with dataset-specific parameters saved in the project’s script folder. For each of the 26 metal-tagged antibody markers, we identified the cellular sub-compartment (cytoplasmic/membrane or nuclear) of interest, based on biological localization of that marker. From there, a single measurement threshold was identified in close collaboration between the image analyst and a pathologist as the cutoff necessary to distinguish positive from negative cells for each marker. This was then validated by the pathologist reading the image through visual observation of the correspondence between presence of those positive cells (above threshold) within the composite panel, relative to the staining intensity of each marker on a per-cell basis. The composite panel containing multiple ROIs and cases helped to refine the selection of this threshold to one value across the entire dataset. Following individual marker thresholds, distinct immune cells of interest were defined as being double or triple positive (co-localization) for their respective characteristic markers as listed in **Supplemental Table S5.** Individual per-cell and per-annotation measurements were extracted from all ROIs and cases studied and exported in .csv format. Prior to any subsequent data analysis, the per-cell statistics file was processed using a separate script to incorporate supplementary patient metadata into the dataset. The per-cell data was represented as a percent positive score by quantifying the percentage of positive cells (positive for either single markers or marker combinations) as an expression of all cells present in each ROI.

The post-image segmentation data analysis for IMC was accomplished using a set of Python scripts. From the raw per-cell and per-marker dataset, the Uniform Manifold Approximation and Projection (UMAP) algorithm^4^ was used to reduce the 26 antibody marker (and Ir193 nuclear counterstain) panel dimensions of the dataset to a two dimensional representation. To enable direct comparison of markers with different staining abundance across the images observed, we applied a Z-score normalization to the data on a per-marker basis prior to fitting into an embedded space for UMAP projection. The UMAP projection, with each point representing an individual cell, was color coded to represent the treatment group to which that cell belonged. A separate panel of UMAP images was generated for each antibody, visualizing the relative localization and intensity range of that marker within the UMAP projection space.

Supplemental Table S1: Criteria for defining AKI-ICI and AKI-Other cohorts

| **AKI was attributed as AKI-ICI by the treating provider and either of the following criteria** | **AKI was attribute as AKI-Other by the treating provider and either of the following criteria** |
| --- | --- |
| **Criteria 1:** Sustained AKI for >48hs  **Criteria 2:** Increase in SCr ≥50% from baseline AND at least one of the following:  1) AIN on kidney biopsy  2) ICI held for at least one cycle due to concern for AKI-ICI  3) Responsiveness to steroids or progression without steroids | **Criteria 1:** Sustained AKI for >48hs  **Criteria 2:** Increase in SCr ≥50% or 0.3 mg/dL from baseline AND at least one of the following  1) Biopsy-confirmed alternative causes  2) Patient did not receive steroids and did not progress. |

Abbreviations: AKI, acute kidney injury; AIN, acute interstitial nephritis; ICI immune checkpoint inhibitor; RRT, renal replacement therapy; SCr, serum creatinine

Supplemental Table S2: Human Enhanced PBMC Phenotyping Panel for Mass Cytometry Analysis

| **Target** | **Tag** | **Antibody Clone** | **Surface or Intracellular** | **Company** | **Catalog No.** | **Dilution Factor** |
| --- | --- | --- | --- | --- | --- | --- |
| CD45 | 089Y | HI30 | Surface | Fluidigm | 3089003B | 400 |
| CD196/CCR6 | 141Pr | G034E3 | Surface | Fluidigm | 3141003A | 200 |
| CD19 | 142Nd | HIB19 | Surface | Fluidigm | 3142001B | 400 |
| CD127/IL-7Ra | 143Nd | A019D5 | Surface | Fluidigm | 3143012B | 200 |
| CD38 | 144Nd | HIT2 | Surface | Fluidigm | 3144014B | 200 |
| CD11a | 145Nd | HI111 | Surface | Biolegend | 301223 | 400 |
| IgD | 146Nd | IA6-2 | Surface | Fluidigm | 3146005B | 400 |
| CD11c | 147Sm | Bu15 | Surface | Fluidigm | 3147008B | 400 |
| CD16 | 148Nd | 3G8 | Surface | Fluidigm | 3148004B | 800 |
| CD194/CCR4 | 149Sm | L291h4 | Surface | Fluidigm | 3149029A | 200 |
| LAG-3 | 150Nd | 11C3C65 | Surface | Fluidigm | 3150030B | 100 |
| CD123/IL-3R | 151Eu | 6H6 | Surface | Fluidigm | 3151001B | 200 |
| TCRgd | 152Sm | 11F2 | Surface | Fluidigm | 3152008B | 400 |
| CD185/CXCR5 | 153Eu | RF8B2 | Surface | Fluidigm | 3153020B | 200 |
| CD3 | 154Sm | UCHT1 | Surface | Fluidigm | 3154003B | 400 |
| CD45RA | 155Gd | HI100 | Surface | Fluidigm | 3155011B | 400 |
| PD-L1 | 156Gd | 29E.2A3 | Surface | Fluidigm | 3156026B | 100 |
| CD27 | 158Gd | L128 | Surface | Fluidigm | 3158010B | 400 |
| Tim-3 | 159Tb | F38-2E2 | Surface | Fluidigm | 3159037C | 100 |
| CD28 | 160Gd | CD28.2 | Surface | Fluidigm | 3160003B | 200 |
| PD-1 | 161Dy | EH12.2H7 | Surface | Biolegend | EH12.2H7 | 100 |
| CD66b | 162Dy | 80H3 | Surface | Fluidigm | 3162023B | 100 |
| CD183/CXCR3 | 163Dy | G025H7 | Surface | Fluidigm | 3163004B | 200 |
| CD161 | 164Dy | HP-3G10 | Surface | Fluidigm | 3164009B | 100 |
| CD45RO | 165Ho | UCHL1 | Surface | Fluidigm | 3165011B | 200 |
| CD24 | 166Er | ML5 | Surface | Fluidigm | 3166007B | 200 |
| CD197/CCR7 | 167Er | G043H7 | Surface | Fluidigm | 3167009A | 200 |
| CD8a | 168Er | SK1 | Surface | Fluidigm | 3168002B | 800 |
| CD25/IL-2R | 169Tm | 2A3 | Surface | Fluidigm | 3169003B | 200 |
| CTLA-4 | 170Er | 14D3 | Surface | Fluidigm | 3170005B | 100 |
| CD20 | 171Yb | 2H7 | Surface | Fluidigm | 3171012B | 800 |
| CX3CR1 | 172Yb | 2A9-1 | Surface | Biolegend | 341602 | 100 |
| HLA-DR | 173Yb | L243 | Surface | Fluidigm | 3173005B | 400 |
| CD4 | 174Yb | SK3 | Surface | Fluidigm | 3174004B | 800 |
| CD14 | 175Lu | M5E2 | Surface | Fluidigm | 3175015B | 200 |
| CD56/NCAM | 176Yb | NCAM16.2 | Surface | Fluidigm | 3176008B | 200 |
| DNA | 191Ir/193Ir |  | Nuclei | Fluidigm | 201192B | 2500 |
| Live/Dead | 195Pt |  | Live/Dead | Fluidigm | 201064 | 10,000 |

Supplemental Table S3. Detection range of the assays

| **Biomarker** | **Detection range (pg/mL)** |
| --- | --- |
| TNF-α | 6.4-100,000 |
| IFN-γ | 1.3-20,000 |
| IL-2 | 0.64-10,000 |
| IL-4 | 0.64-10,000 |
| IL-6 | 0.64-10,000 |
| IL-8 | 0.64-10,000 |
| IL-9 | 0.64-10,000 |
| IL-10 | 2.6-40,000 |

No dilution was performed

Supplemental Table S4. Human Imaging Mass Cytometry Panel

| **Target** | **Tag** | **Antibody Clone** | **Company** | **Catalog No.** | **Dilution Factor** |
| --- | --- | --- | --- | --- | --- |
| Alpha-SMA | 141Pr | 1A4 | Fluidigm | 3141017D | 200 |
| CD19 | 142Nd | 6OMP31 | Fluidigm | 3142014D | 400 |
| Vimentin | 143Nd | D21H3 | Fluidigm | 3143027D | 100 |
| CD14 | 144Nd | EPR3653 | Fluidigm | 3144025D | 200 |
| CD16 | 146Nd | EPR16784 | Fluidigm | 3146020D | 100 |
| Pan-Keratin | 148Nd | C11 | Fluidigm | 3148020D | 200 |
| CD11b | 149Sm | EPR1344 | Fluidigm | 3149028D | 100 |
| CD45 | 152Sm | 2B11 | Fluidigm | 3152016D | 100 |
| CD11c | 154Sm | Polyclonal | Fluidigm | 3154025D | 50 |
| FoxP3 | 155Gd | 236A/E7 | Fluidigm | 3155016D | 50 |
| CD4 | 156Gd | EPR6855 | Fluidigm | 3156033D | 400 |
| E-Cadherin | 158Gd | 24E10 | Fluidigm | 3158029D | 50 |
| CD68 | 159Tb | KP1 | Fluidigm | 3159035D | 100 |
| Vista | 160Gb | D1L2G | Fluidigm | 3160025D | 50 |
| CD20 | 161Dy | H1 | Fluidigm | 3161029D | 800 |
| CD8a | 162Dy | C8/144B | Fluidigm | 3162034D | 100 |
| CD45RA | 166Er | HI100 | Fluidigm | 3166028D | 100 |
| Granzyme B | 167Er | EPR20129-217 | Fluidigm | 3167021D | 50 |
| Ki67 | 168Er | B56 | Fluidigm | 3168022D | 50 |
| Collagen Type 1 | 169Tm | Polyclonal | Fluidigm | 3169023D | 600 |
| CD3 | 170Er | Poly | Fluidigm | 3170019D | 100 |
| Histone H3 | 171Yb | D1H2 | Fluidigm | 3171022D | 600 |
| CD45RO | 173Yb | UCHL1 | Fluidigm | 3173016D | 50 |
| HLA-DR | 174Yb | YE2/36 HLK | Fluidigm | 3174025D | 50 |
| Beta-2 Microglobulin | 175Lu | B2M961 | Abcam | ab212756 | 200 |
| Na/K ATPase | 176Yb | EP1845Y | Abcam | ab283340 | 200 |
| Nuclei | 191Ir/193Ir |  | Fluidigm | 201192B |  |

Supplemental Table S5. Immune cell definitions: Cell types listed in “Immune Cells of Interest” are defined as cells classified as positive for each marker listed in “Positive” (co-localization).

| **Immune Cells of Interest** | **Positive** |
| --- | --- |
| CD4 MEMORY T CELLS | CD4 CD3 CD45RO |
| CD4 NAÏVE T CELL | CD3, CD4, CD45RA |
| CD8 MEMORY T CELLS | CD8 CD3 CD45RO |
| CD8 NAÏVE T CELL | CD3, CD8, CD45RA |
| T HELPER SUBSET | CD3, CD4 |
| T CYTOTOXIC SUBSET | CD3 CD8 |
| T REG CELLS | CD3, CD4, FOXP3 |
| B CELLS | CD19, CD20, |
| DENDRITIC CELLS | CD11C, HLA DR |
| MACROPHAGES | CD11B, CD14, CD68 |
| NK CELLS | CD3, GRANZYME, CD16 |

Supplemental Table S6. Histologic features of n=14 biopsied patients, by cause of AKI

| **Histologic Features, n (%)** | **AKI-ICI**  **(N=10)** | **AKI-Other**  **(N=4)** |
| --- | --- | --- |
| **Acute interstitial nephritis** | 10 (100%) | 0 (0%) |
| **Acute tubular injury** | 9 (90%) | 2 (50%) |
| **Granulomatous features** | 0 (0%) | 0 (0%) |
| **Tissue eosinophilia** | 2 (20%) | 0 (0%) |
| **Tubulitis, moderate to severe** | 5 (50%) | 0 (0%) |
| **Glomerular pathology** | 1 (10%) | 2 (50%) * |
| **Interstitial fibrosis/tubular atrophy** |  |  |
| None/mild | 6 (6%) | 3 (75%) |
| Moderate | 3 (30%) | 1 (25%) |
| **Glomerulosclerosis** |  |  |
| None/mild | 4 (40%) | 4 (100%) |
| **Immunofluorescence microcopy** |  |  |
| **Glomerular deposits** |  |  |
| Minimal mesangial IgM | 0 (0%) | 1 (25%) |
| TBM deposits (IF &EM) | 0 (0%) | 0 (0%) |

Abbreviations: TBM, Tubular basement membrane deposits; IF, Immunofluorescence; EM, electron microscopy.

*Thrombotic microangiopathy

Supplemental Table S7. Correlations between urine cytokines

| **Urine cytokine** |  | **IFN-γ** | **IL-2** | **IL-4** | **IL-6** | **IL-8** | **IL-9** | **IL-10** | **TNF-α** |
| --- | --- | --- | --- | --- | --- | --- | --- | --- | --- |
| IFN-γ | r | 1.00 | 0.26 | 0.43 | 0.10 | 0.43 | -0.04 | 0.20 | 0.26 |
|  | P | - | 0.22 | **0.038** | 0.67 | **0.039** | 0.85 | 0.37 | 0.22 |
| IL-2 | r | 0.26 | 1.00 | -0.16 | 0.07 | 0.39 | 0.32 | 0.39 | 0.31 |
|  | P | 0.22 | - | 0.47 | 0.74 | 0.064 | 0.13 | 0.069 | 0.15 |
| IL-4 | r | 0.43 | -0.16 | 1.00 | -0.14 | -0.18 | -0.25 | 0.09 | -0.03 |
|  | P | **0.038** | 0.47 | - | 0.52 | 0.40 | 0.23 | 0.67 | 0.89 |
| IL-6 | r | 0.10 | 0.07 | -0.14 | 1.00 | 0.45 | -0.08 | 0.13 | 0.24 |
|  | P | 0.67 | 0.74 | 0.52 | - | **0.031** | 0.72 | 0.54 | 0.28 |
| IL-8 | r | 0.43 | 0.39 | -0.18 | 0.45 | 1.00 | 0.02 | 0.27 | 0.47 |
|  | P | **0.039** | 0.064 | 0.40 | **0.031** | - | 0.91 | 0.21 | **0.023** |
| IL-9 | r | -0.04 | 0.32 | -0.25 | -0.08 | 0.02 | 1.00 | 0.01 | 0.24 |
|  | P | 0.85 | 0.13 | 0.23 | 0.72 | 0.91 | - | 0.97 | 0.26 |
| IL-10 | r | 0.20 | 0.39 | 0.09 | 0.13 | 0.27 | 0.01 | 1.00 | 0.81 |
|  | P | 0.37 | 0.069 | 0.67 | 0.54 | 0.21 | 0.97 | - | **<0.001** |
| TNF-α | r | 0.26 | 0.31 | -0.03 | 0.24 | 0.47 | 0.24 | 0.81 | 1.00 |
|  | P | 0.22 | 0.15 | 0.89 | 0.28 | **0.023** | 0.26 | **<0.001** | - |

r=Spearman’s rho.

P-values in bold denote statistical significance at the 0.05 alpha level.

Supplemental Table S8. Plasma cytokines by AKI status

|  | **AKI-ICI (N=14)** | **AKI-Other (N=10)** | **P** |
| --- | --- | --- | --- |
| **INF-γ** | 2.71 (1.27, 4.23) | 1.81 (0.74, 4.05) | 0.64 |
| **IL-2** | 0.68 (0.40, 1.43) | 1.38 (0.37, 2.00) | 0.62 |
| **IL-4** | 0.16 (0.05, 0.72) | 0.12 (0.02, 0.56) | 0.64 |
| **IL-6** | 5.06 (3.44, 9.27) | 6.41 (5.06, 8.37) | 0.53 |
| **IL-8** | 4.91 (3.96, 6.17) | 3.89 (2.24, 13.2) | 0.71 |
| **IL-9** | 6.50 (5.25, 14.9) | 7.17 (4.00, 31.1) | 0.76 |
| **IL-10** | 17.5 (8.62, 24.3) | 12.3 (10.5, 28.2) | 0.97 |
| **TNF-α** | 49.9 (22.8, 71.0) | 52.4 (38.1, 88.1) | 0.44 |

Summary statistics reported are median (IQR). P-values are derived from Wilcoxon rank sum test.

P-values in bold denote statistical significance at the 0.05 alpha level.

Supplemental Table S9. Immune cell phenotype in the peripheral blood, by AKI status

| **Assignment** | **AKI-ICI**  **(N=6)** | **AKI-Other**  **(N=4)** | **P** |
| --- | --- | --- | --- |
| B Cell (Naïve) | 3.23 (2.15, 3.52) | 2.60 (1.36, 4.51) | 0.67 |
| CD4+ T Cell (Central Memory) | 9.36 (6.19, 11.00) | 5.76 (4.38, 6.55) | 0.20 |
| CD4+ T Cell (Effector Memory) | 7.45 (5.62, 9.72) | 6.88 (5.10, 8.33) | 0.83 |
| CD4+ T Cell (EMRA) | 2.46 (0.64, 3.72) | 0.36 (0.16, 0.57) | 0.14 |
| CD4+ T Cell (Naïve) | 3.53 (3.20, 4.02) | 0.48 (0.32, 2.07) | 0.29 |
| CD8+ T Cell (Central Memory) | 1.24 (0.62, 1.56) | 1.73 (1.16, 2.34) | 0.67 |
| CD8+ T Cell (Effector Memory) | 2.69 (1.27, 4.30) | 3.61 (1.40, 6.06) | 0.67 |
| CD8+ T Cell (EMRA) | 1.36 (0.65, 4.15) | 5.32 (0.62, 10.34) | 0.52 |
| CD8+ T Cell (Naïve) | 0.44 (0.22, 0.90) | 1.99 (0.92, 3.25) | 0.20 |
| Dendritic Cell (Plasmacytoid) | 0.08 (0.05, 0.13) | 0.04 (0.03, 0.14) | 0.45 |
| Granulocyte (Basophil) | 0.45 (0.25, 1.06) | 0.35 (0.12, 4.90) | 0.52 |
| Monocyte (CD14+ CD16+) | 2.07 (0.88, 3.61) | 0.89 (0.85, 2.38) | 0.83 |
| NK Cell (CD56+ CD16+) | 3.64 (2.79, 6.36) | 0.81 (0.61, 2.36) | 0.20 |
| NKT Cell | 3.41 (1.11, 5.79) | 0.38 (0.00, 1.91) | 0.087 |

Summary statistics are reported in median (IQR). P-values derived from the Wilcoxon rank sum test.

P-values in bold denote statistical significance at the 0.05 alpha level.

Supplemental Table S10. Immuno cell phenotype in kidney tissue, by AKI status (by single cells markers)

| **Single Immune Cell** | **AKI-ICI**  **(N=4)** | **AKI-Other (N=4)** | **P** |
| --- | --- | --- | --- |
| CD3 | 17.1 (10.3, 22.5) | 2.23 (2.16, 7.81) | 0.15 |
| CD4 | 6.37 (4.20, 9.21) | 0.34 (0.21, 0.61) | **0.021** |
| CD8a | 1.25 (0.62, 1.73) | 0.20 (0.14, 0.25) | 0.25 |
| CD11b | 0.11 (0.05, 0.65) | 0.17 (0.06, 0.82) | 0.66 |
| CD11c | 1.55 (1.15, 2.37) | 0.12 (0.07, 0.22) | **0.043** |
| CD14 | 0.88 (0.52, 3.44) | 0.24 (0.18, 2.00) | 0.56 |
| CD16 | 2.46 (0.96, 5.95) | 1.49 (1.04, 3.63) | 0.77 |
| CD19 | 1.69 (0.38, 3.78) | 1.38 (0.82, 3.46) | 0.56 |
| CD20 | 0.34 (0.13, 0.66) | 0.01 (0.01, 0.03) | 0.15 |
| CD45 | 27.8 (19.5, 34.5) | 5.36 (4.10, 6.69) | **0.021** |
| CD45RO | 6.35 (4.35, 11.74) | 3.55 (2.02, 4.62) | 0.15 |
| CD45RA | 0.41 (0.15, 0.77) | 0.11 (0.05, 0.37) | 0.25 |
| CD68 | 0.64 (0.39, 0.90) | 0.05 (0.04, 0.10) | **0.043** |
| FoxP3 | 5.93 (2.29, 12.41) | 5.84 (4.43, 14.34) | 0.77 |
| HLA.DR | 6.39 (1.35, 14.58) | 0.61 (0.39, 1.05) | 0.083 |
| GranzymeB | 0.10 (0.01, 0.20) | 0.08 (0.05, 0.19) | 0.56 |

Summary statistics are reported in median (IQR). P-values derived from the Wilcoxon rank sum test.

P-values in bold denote statistical significance at the 0.05 alpha level.

**Supplemental Figures:**


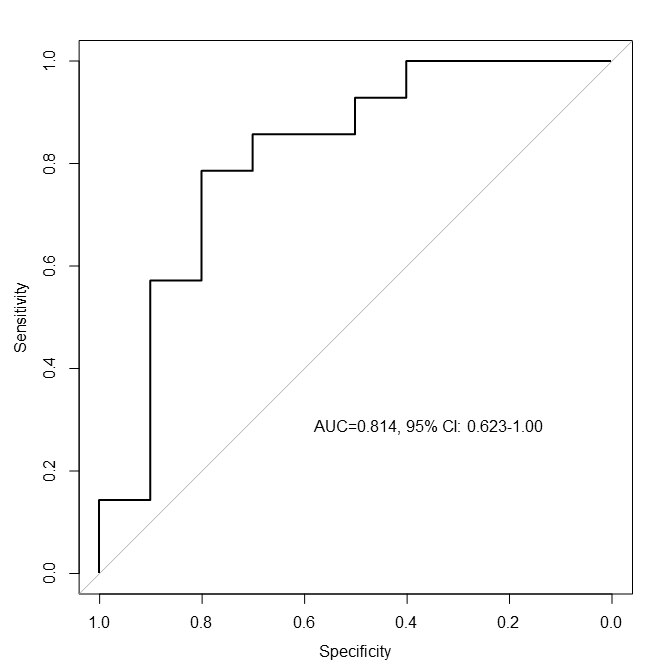


**Supplemental Figure S1:** Receiver operating curve (ROC) for prediction of AKI type (AKI-ICI vs AKI-other) based on urine TNF-α levels. AUC, area under the curve; CI, confidence interval


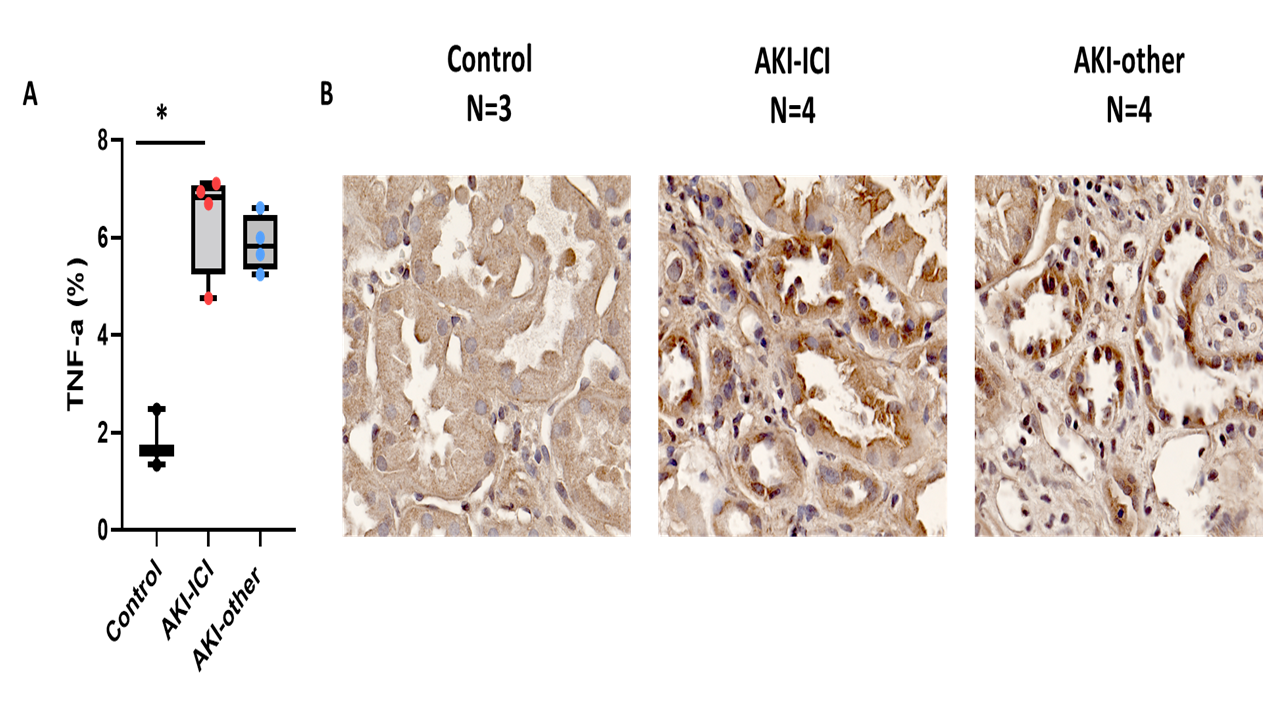


**Supplemental Figure S2:** **TNF-α expression in kidney tissue.** Box & whisker plots of (a) TNF-α expression measure in kidney tissue. The boxes extend from the 25th to the 75th percentile and are bisected by the median; the whiskers extend to the minimum and maximum value . P-values derived from the Kruskal-Wallis test. (b) TNF-α staining of control AKI-ICI and AKI other. AKI, acute kidney injury; ICI, immune checkpoint inhibitors. P-values in bold denote statistical significance at the 0.05 alpha level.

*=P< 0.05 AKI-ICI vs control

**Tables from 3-group comparison**

Supplemental Table S11: Demographic and clinical characteristics, by AKI status and control group

| **Variable** | **AKI-ICI (N=14)** | **AKI-Other (N=10)** | **Controls (N=12)** | **Total (N=36)** | **P** |
| --- | --- | --- | --- | --- | --- |
| **Age (years), mean (SD)** | 65.4 (14.6) | 62.4 (14.2) | 54.2 (9.7) | 60.8 (13.6) | 0.10 |
| **Gender** |  |  |  |  | 0.87 |
| Male | 7 (50.0%) | 6 (60.0%) | 6 (50.0%) | 19 (52.8%) |  |
| Female | 7 (50.0%) | 4 (40.0%) | 6 (50.0%) | 17 (47.2%) |  |
| **White race, n(%)** | 14 (100.0%) | 10 (100.0%) | 11 (91.7%) | 35 (97.2%) | 0.36 |
| **Serum creatinine, median (IQR)** | 1.9 (1.7, 2.4) | 1.7 (1.5, 3.5) | 0.9 (0.8, 1.0) | 1.6 (1.0, 2.2) | **<0.001***† |
| **eGFR (CKD-EPI), mean (SD)** | 29.9 (10.8) | 34.0 (17.9) | 84.9 (11.6) | 49.4 (28.6) | **<0.001***† |

AKI, acute kidney injury; eGFR, estimated glomerular filtration rate; CKD, chronic kidney disease

P-values were derived using the ANOVA test for normally distributed variables, the Kruskal-Wallis test for non-normally distributed variables, the Chi-square test for categorical variables with expected cell counts ≥5, and the Fisher exact test for categorical variables with expected cell counts <5. For post-hoc tests after Kruskal-Wallis, Dunn’s multiple comparison test was used, with p-values adjusted with the Holm method. For post-hoc tests after an ANOVA, Tukey’s test was used. P-values in bold denote statistical significance at the 0.05 alpha level.

*Significant for groups AKI-ICI vs controls in post-hoc test adjusted for multiple comparisons.

†Significant for groups AKI-other vs controls in post-hoc test adjusted for multiple comparisons.

‡ Significant for groups AKI-ICI vs AKI-other in post-hoc test adjusted for multiple comparisons.

Supplemental Table S12. Histologic features of biopsied patients in the AKI-ICI and AKI-other and control group

| **Histologic Features, n (%)** | **AKI-ICI**  **(N=10)** | **AKI-Other**  **(N=4)** | **Controls**  **(N=12)** |
| --- | --- | --- | --- |
| **Acute interstitial nephritis** | 10 (100%) | 0 (0%) | 0 (0%) |
| **Acute tubular injury** | 9 (90%) | 2 (50%) | 0 (0%) |
| **Granulomatous features** | 0 (0%) | 0 (0%) | 0 (0%) |
| **Tissue eosinophilia** | 2 (20%) | 0 (0%) | 0 (0%) |
| **Tubulitis, moderate to severe** | 5 (50%) | 0 (0%) | 0 (0%) |
| **Glomerular pathology** | 1 (10%) | 2 (50%) * | 0 (0%) |
| **Interstitial fibrosis/tubular atrophy** |  |  |  |
| None/mild | 6 (6%) | 3 (75%) | 0 (0%) |
| Moderate | 3 (30%) | 1 (25%) | 0 (0%) |
| **Glomerulosclerosis** |  |  |  |
| None/mild | 4 (40%) | 4 (100%) | 0 (0%) |
| **Immunofluorescence microcopy** |  |  |  |
| **Glomerular deposits** |  |  |  |
| Minimal mesangial IgM | 0 (0%) | 1 (25%) | 0 (0%) |
| TBM deposits (IF &EM) | 0 (0%) | 0 (0%) | 0 (0%) |

Abbreviations: TBM, Tubular basement membrane deposits; IF, Immunofluorescence; EM, electron microscopy.

*Thrombotic microangiopathy

Supplemental Table S13. Urine cytokines, by AKI status and control group

| **Urine cytokine** | **AKI-ICI (N=14)** | **AKI-Other (N=10)** | **Controls (N=12)** | **P** |
| --- | --- | --- | --- | --- |
| **IFN (ng/g)** | 0.63 (0.35, 1.59) | 0.37 (0.08, 0.86) | 0.52 (0.21, 0.94) | 0.51 |
| **IL-2 (ng/g)** | 0.49 (0.35, 0.95) | 0.13 (0.06, 0.39) | 0.14 (0.08, 0.75) | 0.053 |
| **IL-4 (ng/g)** | 0.31 (0.24, 0.50) | 0.36 (0.21, 0.87) | 0.69 (0.43, 0.82) | 0.062 |
| **IL-6 (ng/g)** | 44.8 (20.5, 158) | 30.2 (7.80, 60.9) | 1.42 (0.98, 4.18) | **<0.001***† |
| **IL-8 (ng/g)** | 21.3 (11.1, 85.8) | 20.9 (3.96, 47.8) | 4.28 (2.38, 28.3) | 0.099 |
| **IL-9 (ng/g)** | 6.59 (4.44, 13.4) | 4.65 (2.74, 6.99) | 0.94 (0.72, 4.40) | **0.007*** |
| **IL-10 (ng/g)** | 1.11 (0.87, 1.53) | 0.39 (0.32, 0.65) | 0.50 (0.42, 0.76) | **0.002***‡ |
| **TNF-α (ng/g)** | 4.80 (3.23, 6.89) | 1.95 (1.29, 2.51) | 2.06 (1.56, 2.38) | **0.004***‡ |
| **NGAL (ng/mL)** | 51.7 (29.8, 159) | 48.1 (8.21, 185) | 14.3 (3.32, 17.8) | **0.010*** |
| **KIM-1 (ng/mL)** | 2.02 (1.45, 3.28) | 3.41 (1.00, 4.85) | 0.52 (0.38, 0.80) | **0.002***† |

Summary statistics reported are median (IQR). P-values are derived from Kruskal-Wallis tests. For post-hoc tests, Dunn’s multiple comparison test was used, with p-values adjusted with the Holm method.

*Significant for groups AKI-ICI vs controls in post-hoc test adjusted for multiple comparisons or.

†Significant for groups AKI-other vs controls in post-hoc test adjusted for multiple comparisons.

‡Significant for groups AKI-ICI vs AKI-other in post-hoc test adjusted for multiple comparisons.

P-values in bold denote statistical significance at the 0.05 alpha level.

Supplemental Table S14. Plasma cytokines by AKI status and control group

|  | **AKI-ICI (N=14)** | **AKI-Other (N=10)** | **Controls (N=12)** | **P** |
| --- | --- | --- | --- | --- |
| **INF-γ** | 2.71 (1.27, 4.23) | 1.81 (0.74, 4.05) | 1.18 (0.15, 1.50) | 0.070 |
| **IL-2** | 0.68 (0.40, 1.43) | 1.38 (0.37, 2.00) | 0.51 (0.16, 1.53) | 0.52 |
| **IL-4** | 0.16 (0.05, 0.72) | 0.12 (0.02, 0.56) | 0.09 (0.02, 0.38) | 0.70 |
| **IL-6** | 5.06 (3.44, 9.27) | 6.41 (5.06, 8.37) | 0.56 (0.30, 1.23) | **0.001***† |
| **IL-8** | 4.91 (3.96, 6.17) | 3.89 (2.24, 13.2) | 2.72 (2.18, 3.17) | 0.05 |
| **IL-9** | 6.50 (5.25, 14.9) | 7.17 (4.00, 31.1) | 23.3 (5.56, 48.3) | 0.49 |
| **IL-10** | 17.5 (8.62, 24.3) | 12.3 (10.5, 28.2) | 7.76 (3.60, 13.1) | **0.049** |
| **TNF-α** | 49.9 (22.8, 71.0) | 52.4 (38.1, 88.1) | 43.6 (31.6, 65.4) | 0.66 |

Summary statistics reported are median (IQR). P-values are derived from Kruskal-Wallis tests. For post-hoc tests, Dunn’s multiple comparison test was used, with p-values adjusted with the Holm method

*Significant for groups AKI-ICI vs controls in post-hoc test adjusted for multiple comparisons.

†Significant for groups AKI-other vs controls in post-hoc test adjusted for multiple comparisons.

‡Significant for groups AKI-ICI vs AKI-other in post-hoc test adjusted for multiple comparisons.

P-values in bold denote statistical significance at the 0.05 alpha level.

Supplemental Table S15. Immuno cell phenotype in kidney tissue by AKI status and control group (by single cells markers)

| **Single Immune Cell** | **AKI-ICI**  **(N=4)** | **AKI-Other (N=4)** | **Controls**  **(N=3)** | **P** |
| --- | --- | --- | --- | --- |
| CD3 | 17.1 (10.3, 22.5) | 2.23 (2.16, 7.81) | 0.19 (0.13, 7.43) | 0.18 |
| CD4 | 6.37 (4.20, 9.21) | 0.34 (0.21, 0.61) | 0.03 (0.02, 0.57) | **0.024*** |
| CD8a | 1.25 (0.62, 1.73) | 0.20 (0.14, 0.25) | 0.00 (0.00, 0.29) | 0.18 |
| CD11b | 0.11 (0.05, 0.65) | 0.17 (0.06, 0.82) | 0.05 (0.03, 6.89) | 0.93 |
| CD11c | 1.55 (1.15, 2.37) | 0.12 (0.07, 0.22) | 0.07 (0.04, 0.16) | **0.042** |
| CD14 | 0.88 (0.52, 3.44) | 0.24 (0.18, 2.00) | 1.09 (0.55, 9.99) | 0.79 |
| CD16 | 2.46 (0.96, 5.95) | 1.49 (1.04, 3.63) | 0.12 (0.06, 1.33) | 0.46 |
| CD19 | 1.69 (0.38, 3.78) | 1.38 (0.82, 3.46) | 0.16 (0.08, 0.67) | 0.31 |
| CD20 | 0.34 (0.13, 0.66) | 0.01 (0.01, 0.03) | 0.00 (0.00, 0.02) | 0.13 |
| CD45 | 27.8 (19.5, 34.5) | 5.36 (4.10, 6.69) | 0.43 (0.22, 4.27) | **0.024*** |
| CD45RO | 6.35 (4.35, 11.74) | 3.55 (2.02, 4.62) | 0.13 (0.07, 9.99) | 0.32 |
| CD45RA | 0.41 (0.15, 0.77) | 0.11 (0.05, 0.37) | 0.07 (0.06, 0.20) | 0.32 |
| CD68 | 0.64 (0.39, 0.90) | 0.05 (0.04, 0.10) | 0.03 (0.02, 0.14) | 0.070 |
| FoxP3 | 5.93 (2.29, 12.4) | 5.84 (4.43, 14.3) | 2.01 (1.13, 7.73) | 0.56 |
| HLA.DR | 6.39 (1.35, 14.58) | 0.61 (0.39, 1.05) | 0.39 (0.35, 0.47) | 0.062 |
| GranzymeB | 0.10 (0.01, 0.20) | 0.08 (0.05, 0.19) | 0.10 (0.06, 0.28) | 0.75 |

Summary statistics reported are median (IQR). P-values are derived from Kruskal-Wallis tests. For post-hoc tests, Dunn’s multiple comparison test was used, with p-values adjusted with the Holm method

*Significant for groups AKI-ICI vs controls in post-hoc test adjusted for multiple comparisons.

†Significant for groups AKI-other vs controls in post-hoc test adjusted for multiple comparisons.

‡Significant for groups AKI-ICI vs AKI-other in post-hoc test adjusted for multiple comparisons.

P-values in bold denote statistical significance at the 0.05 alpha level.

**References:**

1. Alexander MP, Mangalaparthi KK, Madugundu AK, et al. Acute Kidney Injury in Severe COVID-19 Has Similarities to Sepsis-Associated Kidney Injury: A Multi-Omics Study. Mayo Clinic proceedings 2021;96:2561-75.

2. Schmidt U, Weigert M, Broaddus C, Myers G. Cell Detection with Star-Convex Polygons. 2018; Cham: Springer International Publishing. p. 265-73.

3. Zaidi M. MT, Wouters B. Universal-StarDist-for-QuPath. 2021.

4. McInnes L, Healy JJA. UMAP: Uniform Manifold Approximation and Projection for Dimension Reduction. 2018;abs/1802.03426.
